# Supplementary material for: Molecular surveillance of zoonotic pathogens from wild rodents in the Republic of Korea
Source: PLoS Negl Trop Dis. 2024 Jul 8;18(7):e0012306. doi: 10.1371/journal.pntd.0012306 (PMC11257403; doi:10.1371/journal.pntd.0012306)
Supplement: S1 Table — (DOCX) [file pntd.0012306.s001.docx]

Supplementary Table 1. Number of captured rodents from each region in each province

| Province | Region | Classification | No. of captured rodents |
| --- | --- | --- | --- |
| Gangwon | Donghae | Peri-urban | 3 |
|  | Jeongseon | Rural | 3 |
|  | Wonju | Rural | 3 |
|  | Yeongwol | Rural | 9 |
| Gyeonggi | Yeoju | Peri-urban | 12 |
| Chungbuk | Cheongju | Peri-urban | 3 |
|  | Chungju | Rural | 5 |
|  | Goesan | Rural | 1 |
|  | Yeongdong | Rural | 10 |
| Chungnam | Geumsan | Rural | 2 |
|  | Daejeon | Peri-urban | 2 |
| Gyeongbuk | Cheongsong | Rural | 6 |
|  | Chilgok | Peri urban | 2 |
|  | Gimcheon | Rural | 6 |
|  | Gumi | Rural | 9 |
|  | Gunwi | Rural | 16 |
|  | Mungyeong | Rural | 5 |
|  | Sangju | Peri-urban | 1 |
|  |  | Rural | 10 |
|  | Uljin | Rural | 3 |
|  | Yecheon | Rural | 2 |
|  | Yeongdeok | Rural | 20 |
| Gyeongnam | Haman | Rural | 4 |
|  | Hapcheon | Rural | 2 |
| Jeonbuk | Buan | Rural | 6 |
|  | Iksan | Rural | 2 |
|  | Muju | Rural | 5 |
| Jeonnam | Yeosu | Peri-urban | 4 |
| Total | | | 156 |
