# Supplementary material for: Molecular surveillance of zoonotic pathogens from wild rodents in the Republic of Korea
Source: PLoS Negl Trop Dis. 2024 Jul 8;18(7):e0012306. doi: 10.1371/journal.pntd.0012306 (PMC11257403; doi:10.1371/journal.pntd.0012306)
Supplement: Attachment — Submitted filename: PNTD-D-23-00918_reviewerA.pdf [file pntd.0012306.s002.pdf]

PLOS Neglected Tropical Diseases

Molecular surveillance of zoonotic pathogens from wild rodents in the Republic of Korea

--Manuscript Draft--

**Manuscript Number:**	PNTD-D-23-00918
**Full Title:**	Molecular surveillance of zoonotic pathogens from wild rodents in the Republic of Korea
**Short Title:**	Dection of rodent-borne pathogens in the Repulic of Korea
**Article Type:**	Research Article
**Keywords:**	Republic of Korea; rodents; rodent-borne pathogens; surveillance; zoonosis
**Abstract:**	Background<br><br>Rodents are recognized as major reservoirs of numerous zoonotic pathogens and are involved in the transmission and maintenance of infectious diseases. Most importantly, despite their importance, diseases transmitted by rodents have been neglected. To date, there have been limited epidemiological studies on rodents and their information is still scarce in the Republic of Korea (ROK).<br><br>Methodology/Principal findings<br><br>We investigated rodent-borne pathogens by PCR/RT-PCR from 156 rodents, including 151 Apodemus agrarius and 5 Rattus norvegicus collected from 27 regions in eight provinces across the ROK between March 2019 and November 2020. Spleen, kidney, and blood samples were used for detecting Anaplasma phagocytophilum, Bartonella spp., Borrelia burgdorferi sensu lato group, Coxiella burnetii, Leptospira interrogans, and severe fever with thrombocytopenia syndrome virus (SFTSV). Of the 156 rodents, 73 (46.8%) were infected with Bartonella spp., 25 (16.0%) with C. burnetii, 24 (15.4%) with L. interrogans, 21 (13.5%) with A. phagocytophilum, 9 (5.8%) with SFTSV, and 5 (3.2%) with Borrelia afzelli. Co-infections with two and three pathogens were detected in 33 (21.1%) and 11 rodents (7.1%), respectively. A. phagocytophilum was detected in all regions, with a widespread occurrence in the ROK. The infection rates of Bartonella spp. were 83.3% for B. grahamii and 16.7% for B. taylorii.<br><br>Conclusions/Significance<br><br>To our best knowledge, this is the first report of C. burnetii and SFTSV infections in rodents in the ROK. Our study also provides the first description of various rodent-borne pathogens through an extensive epidemiological survey in the ROK. Our results suggest that rodents harbor various pathogens, posing a potential threat to public health. Altogether, this study provides useful information on the occurrence and distribution of zoonotic pathogens disseminated among rodents and emphasizes the urgent need for rapid diagnosis, prevention, and control strategies toward these zoonotic diseases.
**Additional Information:**	
**Question**	**Response**
**Government Employee**<br><br>Are you or any of the contributing authors an employee of the United States government?<br><br>Manuscripts authored by one or more US	No - No authors are employees of the U.S. government.

Powered by Editorial Manager® and ProduXion Manager® from Aries Systems Corporation

Government employees are not copyrighted, but are licensed under a CC0 Public Domain Dedication, which allows unlimited distribution and reuse of the article for any lawful purpose. This is a legal requirement for US Government employees.<br><br>This will be typeset if the manuscript is accepted for publication.	
**Financial Disclosure**<br><br>Enter a financial disclosure statement that describes the sources of funding for the work included in this submission. Review the submission guidelines for detailed requirements. View published research articles from *PLOS Neglected Tropical Diseases* for specific examples.<br><br>This statement is required for submission and **will appear in the published article** if the submission is accepted. Please make sure it is accurate.<br><br>**Funded studies**<br>Enter a statement with the following details:<br>• Initials of the authors who received each award<br>• Grant numbers awarded to each author<br>• The full name of each funder<br>• URL of each funder website<br>• Did the sponsors or funders play any role in the study design, data collection and analysis, decision to publish, or preparation of the manuscript?<br><br>Did you receive funding for this work?	Yes
Please add funding details.<br>as follow-up to "**Financial Disclosure**<br><br>Enter a financial disclosure statement that describes the sources of funding for the work included in this submission. Review the submission guidelines for detailed requirements. View published research articles from *PLOS Neglected Tropical*	This study was supported by Kyungpook National University Research Fund, 2022.

Powered by Editorial Manager® and ProduXion Manager® from Aries Systems Corporation

Diseases for specific examples.<br><br>This statement is required for submission and **will appear in the published article** if the submission is accepted. Please make sure it is accurate.<br><br>**Funded studies**<br>Enter a statement with the following details:<br>• Initials of the authors who received each award<br>• Grant numbers awarded to each author<br>• The full name of each funder<br>• URL of each funder website<br>• Did the sponsors or funders play any role in the study design, data collection and analysis, decision to publish, or preparation of the manuscript?<br><br>Did you receive funding for this work?"	
Please select the country of your main research funder (please select carefully as in some cases this is used in fee calculation).<br>as follow-up to "**Financial Disclosure**<br><br>Enter a financial disclosure statement that describes the sources of funding for the work included in this submission. Review the submission guidelines for detailed requirements. View published research articles from *PLOS Neglected Tropical Diseases* for specific examples.<br><br>This statement is required for submission and **will appear in the published article** if the submission is accepted. Please make sure it is accurate.	KOREA, REPUBLIC OF - KR

**Funded studies**<br>Enter a statement with the following details:<br>• Initials of the authors who received each award<br>• Grant numbers awarded to each author<br>• The full name of each funder<br>• URL of each funder website<br>• Did the sponsors or funders play any role in the study design, data collection and analysis, decision to publish, or preparation of the manuscript?<br><br>Did you receive funding for this work?"	
**Competing Interests**<br><br>On behalf of all authors, disclose any competing interests that could be perceived to bias this work.<br><br>This statement will be typeset if the manuscript is accepted for publication.<br><br>Review the instructions link below and PLOS NTDs' competing interests policy to determine what information must be disclosed at submission.	The authors declare that no competing interests exist.
**Data Availability**<br><br>Provide a **Data Availability Statement** in the box below. This statement should detail where the data used in this submission can be accessed. This statement will be typeset if the manuscript is accepted for publication.<br><br>Before publication, authors are required to make all data underlying their findings fully available, without restriction. Review our PLOS Data Policy page for detailed information on this policy. Instructions for writing your Data Availability statement can be accessed via the Instructions link below.	All data generated during this study are included in the article. The nucleotide sequences obtained in the present study have been deposited in the GenBank database under the accession numbers.

Powered by Editorial Manager® and ProduXion Manager® from Aries Systems Corporation

Click here to access/download;Manuscript;PNTD.docx

Molecular surveillance of zoonotic pathogens from wild rodents in the Republic of Korea

Kyoung-Seong Choi[1,*], Sunwoo Hwang[1], Myung Cheol Kim[2], Hyung-Chul Cho[1], Yu-Jin Park[1], Min-Jeong Ji[1], Sun-Woo Han[3], Joon-Seok Chae[3]

[1]Department of Animal Science and Biotechnology, College of Ecology and Environmental Science, Kyungpook National University, Sangju, Republic of Korea

[2]Department of Ecological Science, College of Ecology and Environmental Science, Kyungpook National University, Sangju, Republic of Korea

[3]Laboratory of Veterinary Internal Medicine, BK21 FOUR Future Veterinary Medicine Le ading Education and Research Centre, Research Institute for Veterinary Science and College of Veterinary Medicine, Seoul National University, Seoul, Republic of Korea.

*kschoi3@knu.ac.kr (KSC)

Abstract

Background

Rodents are recognized as major reservoirs of numerous zoonotic pathogens and are involved in the transmission and maintenance of infectious diseases. Most importantly, despite their importance, diseases transmitted by rodents have been neglected. To date, there have been limited epidemiological studies on rodents and their information is still scarce in the Republic of Korea (ROK).

Methodology/Principal findings

We investigated rodent-borne pathogens by PCR/RT-PCR from 156 rodents, including 151 *Apodemus agrarius* and 5 *Rattus norvegicus* collected from 27 regions in eight provinces across the ROK between March 2019 and November 2020. Spleen, kidney, and blood samples were used for detecting *Anaplasma phagocytophilum*, *Bartonella* spp., *Borrelia burgdorferi* sensu lato group, *Coxiella burnetii*, *Leptospira interrogans*, and severe fever with thrombocytopenia syndrome virus (SFTSV). Of the 156 rodents, 73 (46.8%) were infected with *Bartonella* spp., 25 (16.0%) with *C. burnetii*, 24 (15.4%) with *L. interrogans*, 21 (13.5%) with *A. phagocytophilum*, 9 (5.8%) with SFTSV, and 5 (3.2%) with *Borrelia afzelli*. Co-infections with two and three pathogens were detected in 33 (21.1%) and 11 rodents (7.1%), respectively. *A. phagocytophilum* was detected in all regions, with a widespread occurrence in the ROK. The infection rates of *Bartonella* spp. were 83.3% for *B. grahamii* and 16.7% for *B. taylorii*.

Conclusions/Significance

To our best knowledge, this is the first report of *C. burnetii* and SFTSV infections in rodents in the ROK. Our study also provides the first description of various rodent-borne

pathogens through an extensive epidemiological survey in the ROK. Our results suggest that rodents harbor various pathogens, posing a potential threat to public health. Altogether, this study provides useful information on the occurrence and distribution of zoonotic pathogens disseminated among rodents and emphasizes the urgent need for rapid diagnosis, prevention, and control strategies toward these zoonotic diseases.

Author summary

Rodents live anywhere in the world and transmit various infectious diseases to humans and other animals. All the six pathogens examined in this study were detected in rodents. Our findings demonstrated that 66.7% (104/156) of rodents were infected with at least one pathogen. We also observed differences in the pathogens detected in rodents by region. Our results support evidence that rodents play an important role in the transmission of SFTSV. Although we did not screen all rodent-borne diseases, these data will help understand the emerging rodent-borne diseases disseminated in the ROK. These results emphasize the risk of occurrence of rodent-borne diseases.

Introduction

Rodents are globally abundant and well-known reservoirs and vectors of infectious diseases affecting both livestock and humans [1, 2]. The current global change context (e.g., land-use change, urbanization, and temperature increase) is particularly suitable for the expansion of several rodent species beyond their natural distribution areas [3, 4]. Rodents are widespread in rural and urban areas and, in particular, cause numerous human infections in areas where humans are in close contact with rodents. Rodents are reservoir hosts for at least 60 zoonotic diseases and play a vital role in their transmission, which spread directly through contact or bite or indirectly through arthropods [5-7]. Despite their potential threat to public health, there has been less focus on diseases transmitted by rodents [8, 9]. Moreover, the control of rodents is tremendously difficult, considering their behavioral plasticity, life history traits, and high breeding potential [3].

Anaplasma phagocytophilum is a tick-transmitted, obligatory intracellular zoonotic bacterium and infects neutrophils of various hosts, including humans, dogs, cats, horses, domestic animals, and wild animals [10-13]. The clinical signs of *A. phagocytophilum* infection range from asymptomatic to serious symptoms of veterinary and public health importance. The occurrence of *A. phagocytophilum* is increasing along with climate change worldwide. A broad variety of animal species are known to harbor *A. phagocytophilum*, and humans are incidental dead-end hosts [14]. Vertebrate hosts are crucial for the maintenance and circulation of this pathogen in enzootic foci. Of them, in particular, small rodents and wild ruminants have been suggested as primary reservoirs [15-19]. In the United States, the white-footed mouse (*Peromyscus leucopus*) is considered a well-established reservoir species [20, 21]. In the

Republic of Korea (ROK), *A. phagocytophilum* has also been detected in small mammals such as rodents and shrew (*Crocidura lasiura*) [22, 23].

Bartonella spp. are facultative intracellular bacteria that cause persistent infections in erythrocytes and endothelial cells of mammalian hosts [24]. The clinical manifestations caused by these species are characterized by fever, endocarditis, myocarditis, neuroretinitis, lymphadenopathy, and a range of vascular pathologies [24-28]. Currently, more than 30 *Bartonella* spp. and three subspecies are identified [29], and at least 20 species are associated with rodents, indicating that rodents serve as potential reservoirs for zoonotic *Bartonella* spp. [30-32]. Among the rodent adapted *Bartonella* spp., *B. elizabethae*, *B. grahamii*, *B. rochalimae*, *B. tribocorum*, *B. vinsonii*, and *B. washoensis* have been found to cause human infections [32, 33]. In general, *Bartonella* spp. have been considered to be transmitted by arthropods [24, 31]. Although *Bartonella* infections are widely distributed in rodents of different geographic regions [34-41], there is extremely little information on the distribution and prevalence of these species in rodents in the ROK [22, 42, 43].

Lyme borreliosis (LB) is one of the most common vector-borne diseases in North America and Eurasia and caused by a spirochete belonging to the *Borrelia burgdorferi* sensu lato (s.l.) group [44]. Among this group, *B. burgdorferi* sensu stricto (s.s.), *B. afzelii*, and *B. garinii* are the major causative agents of LB in humans and exhibit different geographical distributions [45, 46]. These species are transmitted between vertebrate hosts and tick vectors [47]. *B. burgdorferi* s. s. occurs in North America and Europe and has various reservoir hosts (e.g., rodents and birds), whereas *B. afzelii* and *B. garinii* occur in Eurasia and can only use specific vertebrates such as rodents and birds, respectively [44, 45]. Different *Borrelia* species cause different symptoms in humans. For instance, *B. burgdorferi* s. s. infection is associated with Lyme arthritis, whereas *B. garinii* is mostly linked to neuroborreliosis, and *B. afzelii*

infection is related to a chronic skin condition known as acrodermatitis [44, 48-50]. In the ROK, *B. burgdorferi* s.l. was first detected in 1993 and sporadically identified in ticks, dogs, horses, wild rodents, and humans [51-56].

Coxiella burnetii is an obligate intracellular bacterium with a worldwide distribution and is the causative agent of Q fever in humans and a wide range of animals [57]. It is highly infectious and has the ability to form spore-like particles that withstand harsh environmental conditions and can be easily dispersed by airflow [58]. Humans acquire *C. burnetii* infection through inhalation of contaminated aerosols or dust particles [59]. Q fever is a public health concern as it ranks as one of the 13 leading global priority zoonoses. Moreover, it has been considered a potential biological weapon due to its widespread availability, aerosolized use, and environmental stability [60]. The clinical manifestation of *C. burnetii* infection is characterized by fever and flu-like symptoms. The major sources for these infections are infected ruminants in which the agent may cause abortion and infertility. Ticks and rodents are also known as natural reservoirs of *C. burnetii* [61]. Recently, studies have been conducted on the molecular characterization of this pathogen in domestic animals in the ROK [57, 62]; however, these studies have limited distribution on spatially and are species-specific.

Leptospirosis is a zoonotic infectious disease with a global distribution and is caused by a spirochete of the genus *Leptospira* [63, 64]. It infects more than one million people annually, with 60,000 deaths recorded [65]. *Leptospira* is maintained in several wild and domestic animal hosts through the renal carriage and is excreted in the urine for several months [66, 67]. Infection in humans and animals primarily occurs through direct contact with the urine of infected hosts or indirect exposure to contaminated water, soil, or food [68]. Its clinical manifestations in humans range from mild febrile illness to life-threatening renal failure, pulmonary hemorrhage, and/or cardiac complications [69]. Recent studies suggest that an

increase in the incidence of leptospirosis in humans is often associated with climate changes such as heavy rainfall and flooding [70, 71]. Rodents are considered the most important reservoir of pathogenic *Leptospira* spp. because of their close contact with humans and domestic animals, contributing to disease transmission [72]. *L. interrogans*, *L. borgpetersenii*, and *L. kirschneri* are the most abundant species circulating in humans and animals worldwide [73], with *L. interrogans* being the most described in rodents [72].

Severe fever with thrombocytopenia syndrome (SFTS) is an emerging tick-borne viral disease and has been primarily reported in China, the ROK, Japan, Vietnam, and Taiwan [74-78]. SFTS is caused by *Huaiyangshan banyangvirus* [formerly the SFTS virus (SFTSV)] belonging to the genus *Banyangvirus* in the family *Phenuiviridae*. SFTSV infections are characterized by high fever, fatigue, myalgia, gastrointestinal symptoms, thrombocytopenia, and multiorgan failures [74, 79]. SFTSV could also spread from person to person through exposure to infected blood [80]. Due to the life-threatening threat to public health, SFTS was chosen as one of the nine emerging diseases given a priority for research and development by the World Health Organization in 2017 [81]. As humans are often in close contact with domestic animals and may encounter rodents when they work outdoors, transmission between animals and humans is another possible major transmission route [82]. The overall mortality rate of this disease has been reported to be 3%–30% in different countries [74, 83, 84]. Although SFTSV was identified in various animals, its natural reservoir hosts have not been determined.

As such, rodents are involved in the transmission cycles of various diseases. Recently, the incidence of various infectious diseases is rapidly increasing worldwide due to global warming. Rodent populations are also growing exponentially due to climate change and urbanization. To date, most studies on rodent-borne diseases in the ROK have been primarily

focused on identifying hantavirus infection. Although rodents are considered important reservoirs of zoonotic infectious pathogens, their epidemiological information has been limited in the ROK. Therefore, the aims of this study were to investigate the occurrence of rodent-borne diseases, characterize the genetic relationship, and determine their role as reservoir hosts for these diseases.

Methods

Ethical statement

Rodent collection was approved by the Seoul National University Institutional Animal Care and Use Committee (No. SNU-190524-2-1) and performed according to Seoul National University Guidelines on the care and use of laboratory animals.

Sample collection

Rodents were captured using Sherman traps (3 × 3.5 × 9 inches folding traps; H.B. Sherman Traps, Tallahassee, FL, USA) from 27 regions in eight provinces across the country between March 2019 and November 2020. These traps were set where human infections with SFTSV had been reported based on statistical data of the Korea Disease Control and Prevention Agency. They were installed at regions near rivers, valleys, farms, mountains, and lakes between 5 p.m. and 6 p.m. and retrieved the next day between 9 a.m. and 10 a.m. The captured rodents were transported to the laboratory in an icebox with traps, the species was identified, and they were euthanized using CO_2. Thereafter, blood, spleen, and kidney samples were collected from each animal. A whole blood sample was also collected in an SST, and then serum was separated and used for RNA extraction.

DNA/RNA extraction and PCR analysis

DNA was extracted from spleen (10 mg) and kidney (25 mg) samples using the DNeasy Blood & Tissue Kit (Qiagen, Hilden, Germany) according to the manufacturer's instructions and stored at −20°C until analysis. Splenic DNA was subjected to PCR

amplification to detect *A. phagocytophilum, Bartonella* spp., *Borrelia* spp., and *C. burnetii,* whereas kidney DNA was subjected to detect *L. interrogans*. These pathogens were screened using each specific primer by the nested PCR method under the following conditions: 93°C–95°C for 5 min, followed by 30-40 cycles of 93°C–95°C for 1 min, the annealing temperature of each pathogen, 72°C for 1 min, and a final extension step at 72℃ for 10 min (Table 1). Distilled water was used as a negative control in all PCRs. Secondary PCR products were visualized on 1.5% agarose gels stained with ethidium bromide.

RNA was extracted from 200-μL aliquots of serum using the Gene-spin Viral DNA/RNA Extraction Kit (iNtRON Biotechnology, Seongnam, ROK) according to the manufacturer's instructions. The viral RNA was stored at − 80°C until use. Each RNA sample was tested using nested reverse transcription-polymerase chain reaction (RT-PCR) assays to detect the small (S) segment of SFTSV. Primary PCR was performed using one-step RT-PCR premix (Solgent, Daejeon, ROK) under the following conditions: an initial step of 30 min at 50°C and 15 min at 95°C for denaturation, followed by 40 cycles of 20 s at 95°C, 40 s at 52°C, and 30 s at 72°C, with a final extension step of 5 min at 72°C. Nested PCR was conducted using 1 μL of the primary PCR product as a template (BIOFACT, Daejeon, ROK). The reaction for the nested PCR consisted of 25 cycles of 20 s at 94°C, 40 s at 55 °C, and 30 s at 72°C. The primer information used to detect SFTSV was listed in Table 1. Secondary PCR products were visualized on 1.5% agarose gels stained with ethidium bromide.

Phylogenetic analysis

The secondary PCR products were purified using an AccuPrep® PCR Purification Kit (Bioneer, Daejeon, ROK) according to the manufacturer's instructions and directly sequenced (Macrogen Inc., Seoul, Korea). All the obtained nucleotide sequences for each pathogen were

aligned using the BioEdit software and then compared with reference sequences from the National Center for Biotechnology Information database (http://www.ncbi.nlm.nih.gov) to determine similarity. Phylogenetic analysis of each pathogen was performed using the maximum-likelihood method implemented in MEGA11 using the best substitution model. Bootstrap values were calculated by analyzing 1000 replicates to evaluate the reliability of clusters. The models used in this study were K2 + G for *A. phagocytophilum*, Tamura 3-parameter + G + I for *Bartonella* spp., Tamura-Nei for *Borrelia* spp., and Kimura 2-parameter model for *C. burnetii*, *L. interrogans*, and SFTSV. The nucleotide sequences obtained in this study were assigned the following accession numbers: OR287077–OR287091 for *A. phagocytophilum*, OR288176–OR288190 for *B. grahamii*, OR288191–OR288193 for *B. taylorii*, OR284310–OR284311 for *B. afzelii*, OR284312–OR284321 for *C. burnetii*, OR284322–OR284324 for *L. interrogans*, and OR257718 – OR257726 for SFTSV.

Results

Collection of samples

A total of 175 rodents were captured and morphologically classified as follows: *Apodemus agrarius* (striped field mouse) (*n* = 151), *Rattus norvegicus* (Norway rat) (*n* = 5), and unknown (*n* = 19). Information of the captured rodents was presented in Table 2. Unknown samples were excluded from this study, and the remaining 156 rodents were used for data analysis. *A. agrarius* was mostly found in the ROK, whereas *R. norvegicus* was captured in only two regions.

Prevalence of pathogens detected from captured rodents

The presence of six pathogens was investigated by PCR analysis from the two species, *A. agrarius* and *R. norvegicus*. Of the 156 rodents, 104 (66.7%) were infected with at least one pathogen. None of the tested pathogens were detected in 52 rodents (33.3%). In terms of pathogen, *Bartonella* spp. were the mostly detected (73/156, 46.8%), followed by *C. burnetii* (25/156, 16.0%), *L. interrogans* (24/156, 15.4%), *A. phagocytophilum* (21/156, 13.5%), SFTSV (9/156, 5.8%), and then *Borrelia* spp. (5/156, 3.2%) (Table 3). The details of the pathogens determined according to the regions are shown in Table 3. All six pathogens were detected in Gangwon, Chungbuk, and Gyeongbuk provinces. Five pathogens, except for SFTSV, were found in Gyeongnam province, whereas only one pathogen was detected in Chungnam and Jeonnam provinces (Table 3). Co-infections with two and three pathogens from the captured rodents were also detected in 33 and 11 animals, respectively (Table 4), with co-infection with *Bartonella* spp. and *L. interrogans* being most frequently detected (Table 4). SFTSV was co-infected with *Bartonella* spp. The information of pathogens identified

according to the region is marked in a map (Fig 1). To the best of our knowledge, this is the first study to report C. *burnetii* and SFTSV infections in rodents in the ROK.

Phylogenetic trees of rodent-associated pathogens

Anaplasma phagocytophilum

A. phagocytophilum was detected only in *A. agrarius* and found in all the examined regions, indicating that this pathogen was spread in the ROK. Of the 21 positive samples, 15 were successfully sequenced and confirmed as *A. phagocytophilum* by the phylogenetic tree analysis based on the 16S rRNA gene (Fig 2). Our sequences exhibited 97.6%–99.9% identity to each other and 95.6%–100% identity with those reported from the ROK. The 15 sequences obtained from *A. agrarius* were similar to those previously reported from several different hosts such as cat, cattle, dog, horse, human, tick, and rodents in other countries, sharing 95.9%–100% nucleotide identities with these. Furthermore, several variants co-existed in the same geographical area. According to the phylogenetic tree, *A. phagocytophilum* was divided into clade 1 and clade 2, and all our sequences from *A. agrarius* belonged to clade 1 (Fig 2). The difference in sequences between clade 1 and clade 2 revealed 94.7%–98.5% identities. Clade 2 had 10 nucleotide differences compared with those of clade 1. Genetic variants were detected in *A. phagocytophilum* circulating in the ROK.

Bartonella spp.

Bartonella spp. were the most detected in *A. agrarius* in the ROK, but they were not found in all regions. *Bartonella* spp. were detected in both *A. agrarius* and *R. norvegicus*. Of the 73 ITS PCR-positive samples, 18 sequences were successfully obtained, and all these

originated from *A. agrarius*, not *R. norvegicus* (Table 3). According to the phylogenetic tree based on internal transcribed spacer (*ITS*), *Bartonella* spp. circulating in the examined rodents were identified as two species, viz., *B. grahamii*, and *B. taylorii* (Fig 3). The prevalence of *B. grahamii* was 83.3% (15/18), and that of *B. taylorii* was 16.7% (3/18). The 15 sequences belonging to *B. grahamii* showed 92.9%–100% identity to each other and formed the same group with leeches (KX270012) and another *A. agrarius* (JN810851) reported in the ROK, exhibiting 95.9%–99.8% identity with those. Furthermore, another sequence (JN810855) reported from *A. agrarius* in the ROK demonstrated 87.1%–90.8% similarity to sequences reported in our study. The three sequences classified into *B. taylorii* exhibited 100% identity to each other and shared 92.5%–100% identity with those belonging to this species.

Borrelia **spp.**

Borrelia spp. were detected in five *A. agrarius* and the infection rate of *Borrelia* spp. was the lowest (3.2%) compared with that of other pathogens identified. *Borrelia* spp. were found in four regions (Table 3). Among the five PCR-positive samples, only two sequences were obtained and that demonstrated 98.6% identity to each other. The phylogenetic analysis based on outer surface protein A (*ospA*) gene revealed that our sequences were assigned to *B. afzelii* (Fig 4). The two sequences exhibited 98.9%–100% homology with *A. agrarius* reported previously in the ROK. Our sequences showed 97.8%–100% identity to those belonging to this group. Furthermore, these sequences displayed 98.2%–99.6% similarity to those reported in humans from Austria, Germany, the Czech Republic, Korea, and Sweden.

Coxiella burnetii

C. burnetii was the second most frequently detected pathogen and identified in both *A. agrarius* and *R. norvegicus*. However, it was found in five different regions. Of the 25 positive samples, 10 sequences were obtained and included in the phylogenetic tree based on *IS1111* gene. These sequences showed 97.5%–100% identity to each other. Only one sequence (OR284314) had the closest genetic relationship with those of febrile and pneumonic patients (KP645188 and JF970260), which were known as virulent strains, exhibiting 100% homology with those (Fig 5). The others formed a separate branch, exhibiting 99.0%–99.5% identity to these two human isolates (KP645188 and JF970260). The phylogenetic tree revealed the presence of several genetic clades within *C. burnetii* sequences. These findings indicated the presence of genetic variations in the *C. burnetii* sequences identified in *A. agrarius*.

Leptospira interrogans

L. interrogans was the third most detected pathogen and also found in both *A. agrarius* and *R. norvegicus*. Of the 24 positive samples, only three sequences were obtained and had 97.7%–99.5% identity to each other. The phylogenetic tree based on the RNA polymerase subunit beta (*rpoB*) gene revealed that these sequences belonged to *L. interrogans* (Fig 6). Two sequences (OR284322 and OR284323) were classified into *L. interrogans* serovar *Lai* and showed 99.2%–100% identity with those reported in China and 99.4%–100% identity with *A. agrarius* reported in Korea. The other sequence (OR284324) belonged to *L. interrogans* serovar *Manilae* detected in *Mus musculus* in Japan, exhibiting 98.2% similarity to them (Fig 6). At least two serovars of *L. interrogans* were found to be circulating in *A. agrarius* in the ROK.

Severe fever with thrombocytopenia syndrome virus

SFTSV was detected in nine *A. agrarius* (5.7%) and found in four different regions (Table 3). Of the nine SFTSV infections, single infection of SFTSV was detected only in two *A. agrarius* and the remaining were primarily co-infected with other pathogens such as *Bartonella* spp. and *L. interrogans* (Table 4). Nine sequences were obtained and included in the phylogenetic tree. These sequences demonstrated 95.95%–100.0% identity to each other. The phylogenetic analysis based on the S segments revealed that five and four sequences were classified into subgenotype B-2 and genotype D, respectively (Fig. 7). The sequences belonging to genotype B-2 exhibited 94.51%–97.4% homology with human and other animal samples reported in the ROK, whereas the four sequences showed 99.71%–100.0% identity with human samples. These results revealed that genotype B-2 is prevalent in the ROK, and genetic variants exist within genotype B-2.

Discussion

This study demonstrated the prevalence and genetic characterization of potentially zoonotic pathogens by molecular analysis in rodents captured from throughout the ROK. *A. agrarius* was the most common species in the ROK. Rodents were trapped from areas with frequent movement of people, which may be associated with a high probability of disease transmission because humans and rodents share the same space. All the six pathogens examined in this study were detected in rodents. The results demonstrated that 66.7% (104/156) of rodents were infected with at least one pathogen. According to our findings, *Bartonella* spp. were most frequently detected, and *Borrelia* spp. were least detected in rodents. Although the infection rate was not very high, *A. phagocytophilum* was found in all regions. Considering that the number of rodents captured was different by the region and was small in some provinces, *A. phagocytophilum* may be the most widespread in the ROK. Furthermore, to the best of our knowledge, this is the first study to report *C. burnetii* and SFTSV infections in rodents in the ROK and an extensive study to investigate the infections of various pathogens. ~~Our results demonstrate that rodents play a vital role in the natural infection cycle of *Anaplasma*, *Bartonella*, *Borrelia*, *Coxiella*, *Leptospira*, and SFTSV in the ROK. Therefore, our findings suggest that rodents can directly or indirectly transmit several diseases to humans. Moreover,~~ these data provide valuable information for evaluating the potential risk of rodents in public health.

Anaplasma phagocytophilum has been known as the third most common tick-borne pathogen in the USA and Europe [85] and was detected in 20 different rodent species [86]. *A. phagocytophilum* infection varies considerably in rodent species [86], which may be explained by differences in small mammals that maintain the tick species. In this study, the prevalence of

A. phagocytophilum from *A. agrarius* was 13.5%, which was rather low compared with that reported in a previous study conducted in the ROK (19.1%) [87]. To date, there has been no report of *A. phagocytophilum* infection from *Rattus* spp. in the ROK, although a high infection rate (31.5%) of *A. phagocytophilum* was reported in *Rattus* spp. from China [88]. ~~This suggests that *Rattus norvegicus* is not involved as a reservoir in the transmission cycle of this bacterium in the ROK.~~ *A. phagocytophilum* has been detected in a variety of animals, including ticks in the ROK, but its pathogenicity still remains unclear. ~~When our sequences were compared with those of *A. phagocytophilum* human agent, we observed differences in four of the six nucleotides [89]. According to the phylogenetic analysis, *A. phagocytophilum* circulating in the ROK had several genetic variants. As of now, we cannot conclude whether these variants are pathogenic or non-pathogenic because *A. phagocytophilum* was detected using the 16S rRNA gene. Nevertheless, these variants can infect other hosts as well as humans irrespective of their pathogenicity, and they have been considered zoonotic.~~ *Haemaphysalis longicornis* found primarily in the ROK may tend to use *A. agrarius* as the major host to maintain *A. phagocytophilum*, indicating that *A. agrarius* is an enzootic reservoir. Hence, further studies are required to determine its pathogenicity of *A. phagocytophilum* variants circulating in the ROK.

The overall prevalence of *Bartonella* spp. in *A. agrarius* was 46.8% and the highest compared with that of all other pathogens examined in this study. However, compared with a previous report (62.0%) based on *ITS*, the detection rate in the present study was rather low [43]; this difference may be because of the location where the rodents were captured. Moreover, its prevalence in rodents varied across countries, e.g., 5.5% in Turkey [37], 23.7% in Lithuania [90], 36.3% in Chile [32], 40.4% in Slovenia [34], and 65.8% in Eastern Germany [41]. ~~The difference in prevalence by country may be due to rodent species. Nonetheless, *Bartonella* spp.~~

~~infections are highly prevalent in rodents. Moreover,~~ *Bartonella* spp. that are prevalent in each country are different [27, 32, 34, 38, 90-93]. Although *Bartonella* was detected in both *A. agrarius* and *R. norvegicus*, it was not possible to confirm which species was detected in *R. norvegicus* because the amplified samples from only *A. agrarius* were sequenced. *R. norvegicus* and *R. rattus* have been known as major reservoirs for *Bartonella* spp. in several countries [27, 94-96], but there has been no report of *Bartonella* detection from other rodent species as well as *R. norvegicus* in the ROK [22]. Further studies are necessary to investigate *Bartonella* spp. infection in *R. norvegicus*. The present results demonstrated that *B. grahamii* was most predominant and *B. taylorii* was found in three rodents, a finding consistent with a previous study [43]. *B. grahamii* is a zoonotic pathogen and associated with neuroretinitis and retinal artery occlusion in humans [25]. *B. taylorii* can cause infection in animals [90], but its pathogenicity remains yet unclear. In Europe, *B. taylorii* is dominant in rodents [24, 37]. Although *B. taylorii* has been detected in some *A. agrarius* in the ROK, its transmission route remains unknown. ~~*B. grahamii* identified in this study showed 87.1%–99.8% similarity to those detected previously in the ROK, indicating that genetic diversity exists. At this point, we cannot determine whether the difference is due to host adaptation. Several studies have reported that although the ITS region has high sensitivity in detection, it provides a higher sequential diversity than to other genes [34, 43], which supports our results. Considering the high infection rate in *A. agrarius* and its close contact with humans and other animals, the importance of *Bartonella* as a potential public health concern should not be ignored.~~

The detection rate of *Borrelia* spp. from *A. agrarius* was 3.2% and also the lowest compared with that of other pathogens examined in this study. Our result was different from that of previous studies conducted on heart from *A. agrarius* (29.6%) [56] and in ticks (33.6%) collected from wild rodents [97] in the ROK. This can be explained by the difference in the

sample used. For instance, Kim et al. reported that *B. burgdorferi* s.s. and *B. garinii* infected the spleen and *B. afzelii* exhibited a high detection rate in the heart [56]; however, *B. burgdorferi* s.s. and *B. garinii* were not detected in the spleen. ~~It is speculated that the number of positive samples was small and could not be detected.~~ Among the *Borrelia burgdorferi* s.l. group, only *B. afzelii* was identified in *A. agrarius*, which supports previous findings that *B. afzelii* is the predominant species in the ROK [54, 97]. Furthermore, our results were significantly lower than those reported in rodents from other countries, e.g., 24% in Austria [98], 16% in the Czech Republic [99], and 6.3% in Spain [100]. These differences in prevalence may be due to the tick vectors; the common tick vectors of *Borrelia* spp. in the ROK are *Ixodes persulcatus*, *I. nipponensis*, and *I. granulatus* [101]. *B. afzelii* is transmitted by *Ixodes ricinus* and hosted by small mammals, and it is the most common causative agent of human LB [45, 102]. ~~*B. afzelii* is known to cause acrodermatitis; it readily disseminates from the skin (ear) to joint and heart tissue in its primary natural hosts. The bacterial loads in each tissue differed between host species [44], which may depend on the host species it infects. Collectively, *B. afzelii* possesses the ability to replicate in and attach to a variety of tissues.~~ In the ROK, *B. afzelii* has been primarily reported in ticks [54, 97, 103] and rarely in humans [104]. However, there is still a lack of information on *B. afzelii*. ~~Considering that the infection rate of *B. afzelii* in the ROK is 62.5% in ticks [97] and 25.9% in rodents [56], the possibility that it also occurred in humans cannot be ruled out. In particular, there is very low awareness of the importance of most vector-borne diseases (VBDs) in the ROK, which may have resulted in an underdiagnosis of LB due to similar clinical manifestations of VBDs. Because a high prevalence of *B. afzelii* infection was detected in ticks and rodents, and most importantly, the possibility of LB transmission to humans due to climate change will increase, there exists a need for a systematic strategy for diagnosis, distribution, and control.~~

This is the first report of *C. burnetii* in *A. agrarius* in the ROK. In this study, *C. burnetii* exhibited the second highest infection rate (16%)~~, which was higher than that we anticipated.~~ Nevertheless, our results were lower than those reported in China (18%) [105], Senegal (22.4%) [106], and Zambia (45%) [107], but higher than those reported in Brazil (4.6%) [93], Egypt (6.7%) [58], and Italy (1.4%) [61]. These differences may be explained by the rodent species and samples used for detection. ~~Rodent species, which are predominant, differ in each country.~~ In those studies, *C. burnetii* detection was performed using various samples such as blood, spleen, livers, and feces. Consequently, liver and spleen are considered suitable for the identification of *C. burnetii*. According to a previous study, the infection rate of *C. burnetii* in domestic livestock ranged from 6% to 22.7%, depending on the species [57]. ~~Despite its significance, *C. burnetii* is an underestimated pathogen in the ROK, and there has been no sufficient research on this pathogen.~~ Although *C. burnetii* is a tick-borne pathogen, there are only a few reports of *C. burnetii* in ticks in the ROK [108, 109]. Recent studies have reported about the co-infection of *C. burnetii* and SFTSV in ticks and humans [110, 111]; however, there was no co-infection with two pathogens in rodents. ~~Once *C. burnetii* is detected in rodents, the possibility that *C. burnetii* infection in livestock is transmitted by rodents cannot be ruled out because rodents can frequently enter the barn and infected rodents can contribute to the spread and transmission of this pathogen. Despite the small number of *R. norvegicus* captured, *C. burnetii* infection was mostly detected in *R. norvegicus*, which can be because *R. norvegicus* may also serve as a reservoir in the ROK.~~ A phylogenetic analysis based on *IS1111* gene revealed the presence of two different genotypes within the sequences identified in *A. agrarius*. One sequence formed the same clade with virulent strains reported in Brazil, whereas the others exhibited high similarity to strains reported in different countries. ~~Furthermore, the possibility that the remaining sequences are pathogenic cannot be ignored.~~ The disadvantage of *IS1111*

gene is that it does not provide exact information, such as pathogenicity and species specificity (Fig 5); hence, currently, we cannot draw any conclusions on what separate groupings within *C. burnetii* sequences might represent. Further research is necessary to determine the pathogenicity of *C. burnetii* circulating in the ROK. The results obtained in the present study suggest that *A. agrarius* plays a~~n important~~ role in the transmission of *C. burnetii* in humans and animals.

Leptospira interrogans is a ~~representative~~ rodent-borne pathogen and accordingly, it was the third most frequently detected (15.4%) in this study. Our results demonstrated a relatively high prevalence compared with that of previous studies [87, ~~112]; this difference is due to the regions examined.~~ This is the first time that *Leptospira* has been investigated in rodents through sampling of extensive regions in the ROK. Compared with those reported in other countries, the infection rates ranged from 1.3% to 35.2%, which differed in countries [113-117]. *R. norvegicus* is also an important reservoir of this pathogen [72]; however, *L. interrogans* was detected in only one *R. norvegicus* and mostly detected in *A. agrarius*, which can be due to the limited sample number. ~~Considering that *R. norvegicus* is easily found around barns and farmhouses, it also plays a critical role in the transmission of leptospirosis in domestic animals and humans.~~ To date, *L. interrogans* has been divided into 23 serogroups based on serological methods, with subdivision into more than 300 serovars [72]. The serovars circulating in each country are different, but the most frequently reported serovar worldwide is *Icterohaemorrhagiae* [72]. In the ROK, only a few studies have been conducted on serovar *lai* [87, 118]. Of the three sequences from rodents, two were classified as serovar *lai* and one as serovar *manila*, consistent with a previous study [87]. Consequently, *lai* and *manilae* are considered epidemic serovars in the ROK. However, the biggest limitation of the present study is that a serological analysis such as microscopic agglutination test was not performed, and the

PCR target gene used was also different from that used in other studies. Nonetheless, our results suggest that *rpoB* gene used in this study can be applicable for detection and serovar identification of *L. interrogans*. Furthermore, for an accurate identification of *L. interrogans* serovars, a serological test along with PCR method is absolutely necessary. Leptospirosis has a higher prevalence in tropical or warm-climate countries [72]. Due to global warming, Korea has recently shifted to a subtropical warm and wet climate, and the most representative characteristic is the frequent localized heavy rain, such as flooding. Although there is a lack of sufficient research on leptospirosis in the ROK, the higher incidence observed in the present study than that reported previously may be related to climate change. This provides the opportunity of contamination of rivers or soil and, consequently, the potential risk of leptospirosis. These data highlight the need for prevention and control of leptospirosis.

Since its first identification in China, SFTSV has been primarily detected in Asia [74-78]. Due to its high mortality rate, there is significant interest in SFTSV [74, 83, 84]. In the present study, the infection rate of SFTSV in *A. agrarius* was 5.7%, and this is the first report to describe SFTSV infection from *A. agrarius* in the ROK. Our results were significantly lower than those reported in China (32.3%) [119]. When the infection rates are compared with those in other animals reported in the ROK, the prevalence in rodents was similar to that in wild boars (5.2%) [120] and ticks (6.0%) [121], but higher than that in cats (4.0%) [122], dogs (2.9%) [123], pigs (1.7%) [124], black goats (2.4%) [125], and wild animals (3.3%) [126]. However, the prevalence of SFTSV was highest in feral cats (17.5%) in the ROK [127]. Recently, there is an increase in the populations of feral cats, and they are sharing habitats with wildlife, domestic animals, and humans. Several studies have demonstrated that SFTSV is transmitted to humans through direct contact with cats [128, 129], suggesting that feral cats are infected from rodents. It is believed that SFTSV circulates in a zoonotic cycle between ticks and

vertebrates [130]. Rodents are considered the representative reservoirs in maintaining tick-borne pathogens and may play a vital role in the transmission of SFTSV. ~~Interestingly, in this study, *A. agrarius* was primarily co-infected with *Bartonella* spp. rather than infected with SFTSV alone. As of now, we cannot provide any explanation for the pathogenesis of co-infections. SFTSV can also be transmitted through mouth mucosa or conjunctiva to cause infection [128].~~ The sequences obtained from *A. agrarius* belonged to subgenotype B-2 and D genotype; the results revealed a similar distribution in both genotypes. Sequences belonging to subgenotype B-2 were the most prevalent and associated with the highest mortality rate (43.8%) in the ROK [131], whereas genotype D was primarily found in China. Four sequences belonging to genotype D were identical to those of a human patient reported in the ROK, suggesting that this genotype is pathogenic. Different genotypes of SFTSV are known to trigger different clinical manifestations in a ferret model [130]; however, although clinical manifestations have not been confirmed in rodents, they may be pathogenic to humans. To date, SFTSV has been detected in various animals, but no conclusions could be drawn on how the virus is transmitted to these animals. The results of the present study provide a clue for understanding the transmission route of SFTSV, thereby suggesting the need to establish a continuous monitoring and surveillance system to minimize a serious risk of SFTSV infection.

Conclusions

Urbanization and climate change affect not only on humans but also wildlife. The biggest concern caused by these changes is that the probability of disease transmission through ecosystem destruction has been significantly increasing compared with that in the past. This study investigated the prevalence of zoonotic pathogens in rodent populations through a

systematic epidemiological investigation. Although we did not screen all rodent-borne pathogens, the results indicated that, at least, rodents act as critical reservoirs for *A. phagocytophilum*, *Bartonella* spp., *B. afzelli*, *C. burnetii*, *L. interrogans*, and SFTSV in the ROK. Our findings also demonstrated that rodents harbor several pathogens, implying the possibility of simultaneous transmission to humans. Most importantly, except for SFTSV, the pathogens investigated in this study are misdiagnosed or underdiagnosed in the ROK, so their importance is being neglected. Therefore, our findings indicate that rodents pose a potential risk to public health. Overall, our study provides useful information on rodent-borne pathogens and underscore the urgent need for rapid diagnosis, prevention, and control strategies toward zoonotic diseases.

Acknowledgments

We are very grateful to Dong-Hun Jang, Eun-Mi Kim, and Seung-Uk Shin for their valuable help in sample collection. All authors read and approved the final version.

Author Contributions

KSC and JSC designed the experiments, SH and MCK performed the experiments and sample collection. HCC, YJP, MJJ, and SWH carried out data analysis. KSC wrote the manuscript, which was reviewed by HCC, YJP, MJJ, and JSC.

Data Availability Statement

All data generated during this study are included in the article. The nucleotide sequences obtained in the present study have been deposited in the GenBank database under the accession numbers.

Funding

This study was supported by Kyungpook National University Research Fund, 2022.

Competing interests

The authors declare that no competing interests exist.

References

1. Ecke F, Han BA, Hornfeldt B, Khalil H, Magnusson M, Singh NJ, et al. Population fluctuations and synanthropy explain transmission risk in rodent-borne zoonoses. Nat Commun. 2022;13(1):7532. https://doi.org/10.1038/s41467-022-35273-7. PMID: 36477188.
2. Sumangali K, Rajapakse R, Rajakaruna R. Urban rodents as potential reservoirs of zoonoses: a parasitic survey in two selected areas in Kandy district. Ceylon J Sci. (Bio Sci.) 2012;41(1):71-77. https://doi.org/10.4038/cjsbs.v41i1.4539.
3. Capizzi D, Bertolino S, Mortelliti A. Rating the rat: global patterns and research priorities in impacts and management of rodent pests. Mamm Rev. 2014;44(2):148-162. https://doi.org/10.1111/mam.12019.
4. Dalecky A, Bâ K, Piry S, Lippens C, Diagne CA, Kane M, et al. Range expansion of the invasive house mouse *Mus musculus domesticus* in Senegal, West Africa: a synthesis of trapping data over three decades, 1983–2014. Mamm Rev. 2015;45(3):176-190. https://doi.org/10.1111/mam.12043.
5. Banda A, Gandiwa E, Muposhi VK, Muboko N. Ecological interactions, local people awareness and practices on rodent-borne diseases in Africa: a review. Acta Trop. 2023;238:106743. https://doi.org/10.1016/j.actatropica.2022.106743. PMID: 36343664
6. Lu M, Tang G, Ren Z, Zhang J, Wang W, Qin X, et al. *Ehrlichia*, *Coxiella* and *Bartonella* infections in rodents from Guizhou Province, Southwest China. Ticks Tick Borne Dis. 2022;13(5):101974. https://doi.org/10.1016/j.ttbdis.2022.101974. PMID: 35662068.
7. Meerburg BG, Singleton GR, Kijlstra A. Rodent-borne diseases and their risks for public health. Crit Rev Microbiol. 2009;35(3):221-270. https://doi.org/10.1080/10408410902989837. PMID: 19548807.

8. Morand S, Jittapalapong S, Kosoy M. Rodents as hosts of infectious diseases: biological and ecological characteristics. Vector Borne Zoonotic Dis. 2015;15(1):1-2. https://doi.org/10.1089/vbz.2015.15.1.intro. PMID: 25629774.

9. Selmi R, Belkahia H, Dhibi M, Abdelaali H, Lahmar S, Ben Said M, et al. Zoonotic vector-borne bacteria in wild rodents and associated ectoparasites from Tunisia. Infect Genet Evol. 2021;95:105039. https://doi.org/10.1016/j.meegid.2021.105039. PMID: 34438095.

10. Chen SM, Dumler JS, Bakken JS, Walker DH. Identification of a granulocytotropic Ehrlichia species as the etiologic agent of human disease. J Clin Microbiol. 1994;32(3):589-595. https://doi.org/10.1128/jcm.32.3.589-595.1994. PMID: 8195363.

11. Dumler JS, Barbet AF, Bekker CP, Dasch GA, Palmer GH, Ray SC, et al. Reorganization of genera in the families *Rickettsiaceae* and *Anaplasmataceae* in the order *Rickettsiales*: unification of some species of *Ehrlichia* with *Anaplasma*, *Cowdria* with *Ehrlichia* and *Ehrlichia* with *Neorickettsia*, descriptions of six new species combinations and designation of *Ehrlichia equi* and 'HGE agent' as subjective synonyms of *Ehrlichia phagocytophila*. Int J Syst Evol Microbiol. 2001;51(Pt 6):2145-2165. https://doi.org/10.1099/00207713-51-6-2145. PMID: 11760958.

12. Lappin MR, Breitschwerdt EB, Jensen WA, Dunnigan B, Rha JY, Williams CR, et al. Molecular and serologic evidence of *Anaplasma phagocytophilum* infection in cats in North America. J Am Vet Med Assoc. 2004;225(6):893-896. https://doi.org/10.2460/javma.2004.225.893. PMID: 15485049.

13. Saleem S, Ijaz M, Farooqi SH, Ghaffar A, Ali A, Iqbal K, et al. Equine granulocytic anaplasmosis 28 years later. Microb Pathog. 2018;119:1-8. https://doi.org/10.1016/j.micpath.2018.04.001. PMID: 29626656.

14. Dumler JS, Choi KS, Garcia-Garcia JC, Barat NS, Scorpio DG, Garyu JW, et al. Human

granulocytic anaplasmosis and *Anaplasma phagocytophilum*. Emerg Infect Dis. 2005;11(12):1828-1834. https://doi.org/10.3201/eid1112.050898. PMID: 16485466.

15. Nicholson WL, Muir S, Sumner JW, Childs JE. Serologic evidence of infection with *Ehrlichia* spp. in wild rodents (Muridae: Sigmodontinae) in the United States. J Clin Microbiol. 1998;36(3):695-700. https://doi.org/10.1128/JCM.36.3.695-700.1998. PMID: 9508298.
16. Alberdi MP, Walker AR, Urquhart KA. Field evidence that roe deer (*Capreolus capreolus*) are a natural host for *Ehrlichia phagocytophila*. Epidemiol Infect. 2000;124(2):315-323. https://doi.org/10.1017/s0950268899003684. PMID: 10813158.
17. Bown KJ, Lambin X, Telford GR, Ogden NH, Telfer S, Woldehiwet Z, et al. Relative importance of *Ixodes ricinus* and *Ixodes trianguliceps* as vectors for *Anaplasma phagocytophilum* and *Babesia microti* in field vole (*Microtus agrestis*) populations. Appl Environ Microbiol. 2008;74(23):7118-7125. https://doi.org/10.1128/AEM.00625-08. PMID: 18820068.
18. Petrovec M, Sixl W, Schweiger R, Mikulasek S, Elke L, Wust G, et al. Infections of wild animals with *Anaplasma phagocytophila* in Austria and the Czech Republic. Ann N Y Acad Sci. 2003;990:103-106. https://doi.org/10.1111/j.1749-6632.2003.tb07345.x. PMID: 12860608.
19. Scharf W, Schauer S, Freyburger F, Petrovec M, Schaarschmidt-Kiener D, Liebisch G, et al. Distinct host species correlate with *Anaplasma phagocytophilum ankA* gene clusters. J Clin Microbiol. 2011;49(3):790-796. https://doi.org/10.1128/JCM.02051-10. PMID: 21177886.
20. Ravyn MD, Kodner CB, Carter SE, Jarnefeld JL, Johnson RC. Isolation of the etiologic agent of human granulocytic ehrlichiosis from the white-footed mouse (*Peromyscus*

leucopus). J Clin Microbiol. 2001;39(1):335-338. https://doi.org/10.1128/JCM.39.1.335-338.2001. PMID: 11136794.

21. Telford SR, 3rd, Dawson JE. Persistent infection of C3H/HeJ mice by *Ehrlichia chaffeensis*. Vet Microbiol. 1996;52(1-2):103-112. https://doi.org/10.1016/0378-1135(96)00064-8. PMID: 8914255.
22. Chae JS, Yu DH, Shringi S, Klein TA, Kim HC, Chong ST, et al. Microbial pathogens in ticks, rodents and a shrew in northern Gyeonggi-do near the DMZ, Korea. J Vet Sci. 2008;9(3):285-293. https://doi.org/10.4142/jvs.2008.9.3.285. PMID: 18716449.
23. Kim CM, Yi YH, Yu DH, Lee MJ, Cho MR, Desai AR, et al. Tick-borne rickettsial pathogens in ticks and small mammals in Korea. Appl Environ Microbiol. 2006;72(9):5766-5776. https://doi.org/10.1128/AEM.00431-06. PMID: 16957192.
24. Tolkacz K, Alsarraf M, Kowalec M, Dwuznik D, Grzybek M, Behnke JM, et al. *Bartonella* infections in three species of *Microtus*: prevalence and genetic diversity, vertical transmission and the effect of concurrent *Babesia microti* infection on its success. Parasit Vectors. 2018;11(1):491. https://doi.org/10.1186/s13071-018-3047-6. PMID: 30165879.
25. Kerkhoff FT, Bergmans AM, van Der Zee A, Rothova A. Demonstration of *Bartonella grahamii* DNA in ocular fluids of a patient with neuroretinitis. J Clin Microbiol. 1999;37(12):4034-4038. https://doi.org/10.1128/JCM.37.12.4034-4038.1999. PMID: 10565926.
26. Kosoy M, Murray M, Gilmore RD, Jr., Bai Y, Gage KL. *Bartonella* strains from ground squirrels are identical to *Bartonella washoensis* isolated from a human patient. J Clin Microbiol. 2003;41(2):645-650. https://doi.org/10.1128/JCM.41.2.645-650.2003. PMID: 12574261.
27. Krugel M, Pfeffer M, Krol N, Imholt C, Baert K, Ulrich RG, et al. Rats as potential

reservoirs for neglected zoonotic *Bartonella* species in flanders, Belgium. Parasit Vectors. 2020;13(1):235. https://doi.org/10.1186/s13071-020-04098-y. PMID: 32381113.

28. Oksi J, Rantala S, Kilpinen S, Silvennoinen R, Vornanen M, Veikkolainen V, et al. Cat scratch disease caused by *Bartonella grahamii* in an immunocompromised patient. J Clin Microbiol. 2013;51(8):2781-2784. https://doi.org/10.1128/JCM.00910-13. PMID: 23740723.
29. Okaro U, Addisu A, Casanas B, Anderson B. *Bartonella* species, an emerging cause of blood-culture-negative endocarditis. Clin Microbiol Rev. 2017;30(3):709-746. https://doi.org/10.1128/CMR.00013-17. PMID: 28490579.
30. Goncalves LR, Favacho AR, Roque AL, Mendes NS, Fidelis Junior OL, Benevenute JL, et al. Association of *Bartonella* species with wild and synanthropic rodents in different Brazilian Biomes. Appl Environ Microbiol. 2016;82(24):7154-7164. https://doi.org/10.1128/AEM.02447-16. PMID: 27736785.
31. Gutierrez R, Krasnov B, Morick D, Gottlieb Y, Khokhlova IS, Harrus S. *Bartonella* infection in rodents and their flea ectoparasites: an overview. Vector Borne Zoonotic Dis. 2015;15(1):27-39. https://doi.org/10.1089/vbz.2014.1606. PMID: 25629778.
32. Muller A, Gutierrez R, Seguel M, Monti G, Otth C, Bittencourt P, et al. Molecular survey of *Bartonella* spp. in rodents and fleas from Chile. Acta Trop. 2020;212:105672. https://doi.org/10.1016/j.actatropica.2020.105672. PMID: 32835672.
33. Buffet JP, Kosoy M, Vayssier-Taussat M. Natural history of *Bartonella*-infecting rodents in light of new knowledge on genomics, diversity and evolution. Future Microbiol. 2013;8(9):1117-1128. https://doi.org/10.2217/fmb.13.77. PMID: 24020740.
34. Knap N, Duh D, Birtles R, Trilar T, Petrovec M, Avsic-Zupanc T. Molecular detection of *Bartonella* species infecting rodents in Slovenia. FEMS Immunol Med Microbiol.

2007;50(1):45-50. https://doi.org/10.1111/j.1574-695X.2007.00226.x. PMID: 17374132.

35. Kosoy MY, Regnery RL, Tzianabos T, Marston EL, Jones DC, Green D, et al. Distribution, diversity, and host specificity of *Bartonella* in rodents from the southeastern United States. Am J Trop Med Hyg. 1997;57(5):578-588. https://doi.org/10.4269/ajtmh.1997.57.578. PMID: 9392599.

36. Pretorius AM, Beati L, Birtles RJ. Diversity of bartonellae associated with small mammals inhabiting free state province, South Africa. Int J Syst Evol Microbiol. 2004;54(Pt 6):1959-1967. https://doi.org/10.1099/ijs.0.03033-0. PMID: 15545418.

37. Polat C, Celebi B, Irmak S, Karatas A, Colak F, Matur F, et al. Characterization of *Bartonella taylorii* strains in small mammals of the Turkish Thrace. Ecohealth. 2020;17(4):477-486. https://doi.org/10.1007/s10393-021-01518-y. PMID: 33788082.

38. Qin XR, Liu JW, Yu H, Yu XJ. *Bartonella* species detected in rodents from eastern China. Vector Borne Zoonotic Dis. 2019;19(11):810-814. https://doi.org/10.1089/vbz.2018.2410. PMID: 31355717.

39. Rodriguez-Pastor R, Escudero R, Lambin X, Vidal MD, Gil H, Jado I, et al. Zoonotic pathogens in fluctuating common vole (*Microtus arvalis*) populations: occurrence and dynamics. Parasitology. 2019;146(3):389-398. https://doi.org/10.1017/S0031182018001543. PMID: 30246665.

40. Rothenburger JL, Himsworth CG, Nemeth NM, Pearl DL, Jardine CM. Beyond abundance: How microenvironmental features and weather influence *Bartonella tribocorum* infection in wild Norway rats (*Rattus norvegicus*). Zoonoses Public Health. 2018;65(3):339-351. https://doi.org/10.1111/zph.12440. PMID: 29274119.

41. Silaghi C, Pfeffer M, Kiefer D, Kiefer M, Obiegala A. *Bartonella*, rodents, fleas and ticks: a molecular field study on host-vector-pathogen associations in Saxony, Eastern Germany.

Microb Ecol. 2016;72(4):965-974. https://doi.org/10.1007/s00248-016-0787-8. PMID: 27220973.

42. Kim CM, Kim JY, Yi YH, Lee MJ, Cho MR, Shah DH, et al. Detection of *Bartonella* species from ticks, mites and small mammals in Korea. J Vet Sci. 2005;6(4):327-334. PMID: 16293997.
43. Ko S, Kang JG, Kim HC, Klein TA, Choi KS, Song JW, et al. Prevalence, isolation and molecular characterization of *Bartonella* species in Republic of Korea. Transbound Emerg Dis. 2016;63(1):56-67. https://doi.org/10.1111/tbed.12217. PMID: 24661833.
44. Zhong X, Nouri M, Raberg L. Colonization and pathology of *Borrelia afzelii* in its natural hosts. Ticks Tick Borne Dis. 2019;10(4):822-827. https://doi.org/10.1016/j.ttbdis.2019.03.017. PMID: 31005618.
45. Kurtenbach K, Hanincova K, Tsao JI, Margos G, Fish D, Ogden NH. Fundamental processes in the evolutionary ecology of Lyme borreliosis. Nat Rev Microbiol. 2006;4(9):660-669. https://doi.org/10.1038/nrmicro1475. PMID: 16894341.
46. Stanek G, Reiter M. The expanding Lyme *Borrelia* complex--clinical significance of genomic species? Clin Microbiol Infect. 2011;17(4):487-493. https://doi.org/10.1111/j.1469-0691.2011.03492.x. PMID: 21414082.
47. Rollins RE, Yeyin Z, Wyczanska M, Alig N, Hepner S, Fingerle V, et al. Spatial variability in prevalence and genospecies distributions of *Borrelia burgdorferi* sensu lato from ixodid ticks collected in southern Germany. Ticks Tick Borne Dis. 2021;12(1):101589. https://doi.org/10.1016/j.ttbdis.2020.101589. PMID: 33096511.
48. Balmelli T, Piffaretti JC. Association between different clinical manifestations of Lyme disease and different species of *Borrelia burgdorferi* sensu lato. Res Microbiol. 1995;146(4):329-340. https://doi.org/10.1016/0923-2508(96)81056-4. PMID: 7569327.

49. Wang G, van Dam AP, Schwartz I, Dankert J. Molecular typing of *Borrelia burgdorferi* sensu lato: taxonomic, epidemiological, and clinical implications. Clin Microbiol Rev. 1999;12(4):633-653. https://doi.org/10.1128/CMR.12.4.633. PMID: 10515907.
50. Scheffold N, Herkommer B, Kandolf R, May AE. Lyme carditis--diagnosis, treatment and prognosis. Dtsch Arztebl Int. 2015;112(12):202-208. https://doi.org/10.3238/arztebl.2015.0202. PMID: 25838022.
51. Kee S, Hwang KJ, Oh HB, Park KS. Identification of *Borrelia burgdorferi* isolated in Korea using outer surface protein A (*OspA*) serotyping system. Microbiol Immunol. 1994;38(12):989-993. https://doi.org/10.1111/j.1348-0421.1994.tb02157.x. PMID: 7723693.
52. Bell DR, Berghaus RD, Patel S, Beavers S, Fernandez I, Sanchez S. Seroprevalence of tick-borne infections in military working dogs in the Republic of Korea. Vector Borne Zoonotic Dis. 2012;12(12):1023-1030. https://doi.org/10.1089/vbz.2011.0864. PMID: 23167501.
53. Lee SH, Yun SH, Choi E, Park YS, Lee SE, Cho GJ, et al. Serological detection of *Borrelia burgdorferi* among horses in Korea. Korean J Parasitol. 2016;54(1):97-101. https://doi.org/10.3347/kjp.2016.54.1.97. PMID: 26951987.
54. VanBik D, Lee SH, Seo MG, Jeon BR, Goo YK, Park SJ, et al. *Borrelia* species detected in ticks feeding on wild Korean Water Deer (*Hydropotes inermis*) using molecular and genotypic analyses. J Med Entomol. 2017;54(5):1397-1402. https://doi.org/10.1093/jme/tjx106. PMID: 28549151.
55. Moon S, Hong Y, Hwang KJ, Kim S, Eom J, Kwon D, et al. Epidemiological features and clinical manifestations of Lyme borreliosis in Korea during the period 2005-2012. Jpn J Infect Dis. 2015;68(1):1-4. https://doi.org/10.7883/yoken.JJID.2013.462. PMID:

25420645.

56. Kim CM, Park SY, Kim DM, Park JW, Chung JK. First report of *Borrelia burgdorferi* sensu stricto detection in a commune genospecies in *Apodemus agrarius* in Gwangju, South Korea. Sci Rep. 2021;11(1):18199. https://doi.org/10.1038/s41598-021-97411-3. PMID: 34521873.
57. Cho HC, Hwang S, Kim EM, Park YJ, Shin SU, Jang DH, et al. Prevalence and molecular characterization of *Coxiella burnetii* in cattle, goats, and horses in the Republic of Korea. Vector Borne Zoonotic Dis. 2021;21(7):502-508. https://doi.org/10.1089/vbz.2020.2764. PMID: 33844947.
58. Abdel-Moein KA, Hamza DA. Rat as an overlooked reservoir for *Coxiella burnetii*: a public health implication. Comp Immunol Microbiol Infect Dis. 2018;61:30-33. https://doi.org/10.1016/j.cimid.2018.11.002. PMID: 30502830.
59. Chochlakis D, Santos AS, Giadinis ND, Papadopoulos D, Boubaris L, Kalaitzakis E, et al. Genotyping of *Coxiella burnetii* in sheep and goat abortion samples. BMC Microbiol. 2018;18(1):204. https://doi.org/10.1186/s12866-018-1353-y. PMID: 30514233.
60. Madariaga MG, Rezai K, Trenholme GM, Weinstein RA. Q fever: a biological weapon in your backyard. Lancet Infect Dis. 2003;3(11):709-721. https://doi.org/10.1016/s1473-3099(03)00804-1. PMID: 14592601.
61. Pascucci I, Di Domenico M, Dall'Acqua F, Sozio G, Camma C. Detection of lyme disease and Q fever agents in wild rodents in central Italy. Vector Borne Zoonotic Dis. 2015;15(7):404-411. https://doi.org/10.1089/vbz.2015.1807. PMID: 26134933.
62. Hwang S, Cho HC, Shin SU, Kim HY, Park YJ, Jang DH, et al. Seroprevalence and molecular characterization of *Coxiella burnetii* in cattle in the Republic of Korea. Pathogens. 2020;9(11):890. https://doi.org/10.3390/pathogens9110890. PMID: 33121031.

63. Esteves LM, Bulhoes SM, Branco CC, Carreira T, Vieira ML, Gomes-Solecki M, et al. Diagnosis of human leptospirosis in a clinical setting: real-time PCR high resolution melting analysis for detection of *Leptospira* at the onset of disease. Sci Rep. 2018;8(1):9213. https://doi.org/10.1038/s41598-018-27555-2. PMID: 29907838.
64. Karpagam KB, Ganesh B. Leptospirosis: a neglected tropical zoonotic infection of public health importance-an updated review. Eur J Clin Microbiol Infect Dis. 2020;39(5):835-846. https://doi.org/10.1007/s10096-019-03797-4. PMID: 31898795.
65. Lopez-Osorio S, Molano DA, Lopez-Arias A, Rodriguez-Osorio N, Zambrano C, Chaparro-Gutierrez JJ. Seroprevalence and molecular characterization of *Leptospira* spp. in rats captured near pig farms in Colombia. Int J Environ Res Public Health. 2022;19(18):11539. https://doi.org/10.3390/ijerph191811539. PMID: 36141812.
66. Bharti AR, Nally JE, Ricaldi JN, Matthias MA, Diaz MM, Lovett MA, et al. Leptospirosis: a zoonotic disease of global importance. Lancet Infect Dis. 2003;3(12):757-771. https://doi.org/10.1016/s1473-3099(03)00830-2. PMID: 14652202.
67. Ko AI, Goarant C, Picardeau M. *Leptospira*: the dawn of the molecular genetics era for an emerging zoonotic pathogen. Nat Rev Microbiol. 2009;7(10):736-747. https://doi.org/10.1038/nrmicro2208. PMID: 19756012.
68. Ellis WA. Animal leptospirosis. Curr Top Microbiol Immunol. 2015;387:99-137. https://doi.org/10.1007/978-3-662-45059-8_6. PMID: 25388134.
69. Levett PN. Leptospirosis. Clin Microbiol Rev. 2001;14(2):296-326. https://doi.org/10.1128/CMR.14.2.296-326.2001. PMID: 11292640.
70. Lau CL, Smythe LD, Craig SB, Weinstein P. Climate change, flooding, urbanisation and leptospirosis: fuelling the fire? Trans R Soc Trop Med Hyg. 2010;104(10):631-638. https://doi.org/10.1016/j.trstmh.2010.07.002. PMID: 20813388.

71. Schonning MH, Phelps MD, Warnasekara J, Agampodi SB, Furu P. A case-control study of environmental and occupational risks of leptospirosis in Sri Lanka. Ecohealth. 2019;16(3):534-543. https://doi.org/10.1007/s10393-019-01448-w. PMID: 31664587.
72. Boey K, Shiokawa K, Rajeev S. *Leptospira* infection in rats: a literature review of global prevalence and distribution. PLoS Negl Trop Dis. 2019;13(8):e0007499. https://doi.org/10.1371/journal.pntd.0007499. PMID: 31398190.
73. Thaipadungpanit J, Wuthiekanun V, Chierakul W, Smythe LD, Petkanchanapong W, Limpaiboon R, et al. A dominant clone of *Leptospira interrogans* associated with an outbreak of human leptospirosis in Thailand. PLoS Negl Trop Dis. 2007;1(1):e56. https://doi.org/10.1371/journal.pntd.0000056. PMID: 17989782.
74. Yu XJ, Liang MF, Zhang SY, Liu Y, Li JD, Sun YL, et al. Fever with thrombocytopenia associated with a novel bunyavirus in China. N Engl J Med. 2011;364(16):1523-1532. https://doi.org/10.1056/NEJMoa1010095. PMID: 21410387.
75. Kim KH, Yi J, Kim G, Choi SJ, Jun KI, Kim NH, et al. Severe fever with thrombocytopenia syndrome, South Korea, 2012. Emerg Infect Dis. 2013;19(11):1892-1894. https://doi.org/10.3201/eid1911.130792. PMID: 24206586.
76. Takahashi T, Maeda K, Suzuki T, Ishido A, Shigeoka T, Tominaga T, et al. The first identification and retrospective study of severe fever with thrombocytopenia syndrome in Japan. J Infect Dis. 2014;209(6):816-827. https://doi.org/10.1093/infdis/jit603. PMID: 24231186.
77. Tran XC, Yun Y, Van An L, Kim SH, Thao NTP, Man PKC, et al. Endemic severe fever with thrombocytopenia syndrome, Vietnam. Emerg Infect Dis. 2019;25(5):1029-1031. https://doi.org/10.3201/eid2505.181463. PMID: 31002059.
78. Lin TL, Ou SC, Maeda K, Shimoda H, Chan JP, Tu WC, et al. The first discovery of severe

fever with thrombocytopenia syndrome virus in Taiwan. Emerg Microbes Infect. 2020;9(1):148-151. https://doi.org/10.1080/22221751.2019.1710436. PMID: 31918622.

79. Liu JW, Zhao L, Luo LM, Liu MM, Sun Y, Su X, et al. Molecular evolution and spatial transmission of severe fever with thrombocytopenia syndrome virus based on complete cenome sequences. PLoS One. 2016;11(3):e0151677. https://doi.org/10.1371/journal.pone.0151677. PMID: 26999664.

80. Bao CJ, Guo XL, Qi X, Hu JL, Zhou MH, Varma JK, et al. A family cluster of infections by a newly recognized bunyavirus in eastern China, 2007: further evidence of person-to-person transmission. Clin Infect Dis. 2011;53(12):1208-1214. https://doi.org/10.1093/cid/cir732. PMID: 22028437.

81. Mehand MS, Millett P, Al-Shorbaji F, Roth C, Kieny MP, Murgue B. World health organization methodology to prioritize emerging infectious diseases in need of research and development. Emerg Infect Dis. 2018;24(9):e171427. https://doi.org/10.3201/eid2409.171427. PMID: 30124424.

82. Cheng J, Zhang L, Hu B, Wang Q, Wu R, Zhan F, et al. Prevalence and molecular phylogenetic analysis of severe fever with thrombocytopenia syndrome virus in domestic animals and rodents in Hubei Province, China. Virol Sin. 2019;34(5):596-600. https://doi.org/10.1007/s12250-019-00119-y. PMID: 31161553.

83. Choi SJ, Park SW, Bae IG, Kim SH, Ryu SY, Kim HA, et al. Severe fever with thrombocytopenia syndrome in South Korea, 2013-2015. PLoS Negl Trop Dis. 2016;10(12):e0005264. https://doi.org/10.1371/journal.pntd.0005264. PMID: 28033338.

84. Kobayashi Y, Kato H, Yamagishi T, Shimada T, Matsui T, Yoshikawa T, et al. Severe fever with thrombocytopenia syndrome, Japan, 2013-2017. Emerg Infect Dis. 2020;26(4):692-699. https://doi.org/10.3201/eid2604.191011. PMID: 32186502.

85. Dumler JS. The biological basis of severe outcomes in *Anaplasma phagocytophilum* infection. FEMS Immunol Med Microbiol. 2012;64(1):13-20. https://doi.org/10.1111/j.1574-695X.2011.00909.x. PMID: 22098465.

86. Stuen S, Granquist EG, Silaghi C. *Anaplasma phagocytophilum*--a widespread multi-host pathogen with highly adaptive strategies. Front Cell Infect Microbiol. 2013;3:31. https://doi.org/10.3389/fcimb.2013.00031. PMID: 23885337.

87. Bang MS, Kim CM, Park JW, Chung JK, Kim DM, Yun NR. Prevalence of *Orientia tsutsugamushi*, *Anaplasma phagocytophilum* and *Leptospira interrogans* in striped field mice in Gwangju, Republic of Korea. PLoS One. 2019;14(8):e0215526. https://doi.org/10.1371/journal.pone.0215526. PMID: 31419222.

88. Zheng W, Liu Y, Tao H, Li Z, Xuan X, Liu X, et al. First Molecular evidence of *Anaplasma phagocytophilum* in rodent populations of Nanchang, China. Jpn J Infect Dis. 2018;71(2):129-133. https://doi.org/10.7883/yoken.JJID.2017.301. PMID: 29491242.

89. Massung RF, Mauel MJ, Owens JH, Allan N, Courtney JW, Stafford KC, 3rd, et al. Genetic variants of *Ehrlichia phagocytophila*, Rhode Island and Connecticut. Emerg Infect Dis. 2002;8(5):467-472. https://doi.org/10.3201/eid0805.010251. PMID: 11996680.

90. Lipatova I, Paulauskas A, Puraite I, Radzijevskaja J, Balciauskas L, Gedminas V. *Bartonella* infection in small mammals and their ectoparasites in Lithuania. Microbes Infect. 2015;17(11-12):884-888. https://doi.org/10.1016/j.micinf.2015.08.013. PMID: 26344603.

91. Pangjai D, Maruyama S, Boonmar S, Kabeya H, Sato S, Nimsuphan B, et al. Prevalence of zoonotic *Bartonella* species among rodents and shrews in Thailand. Comp Immunol Microbiol Infect Dis. 2014;37(2):109-114. https://doi.org/10.1016/j.cimid.2013.12.001. PMID: 24393304.

92. Malania L, Bai Y, Osikowicz LM, Tsertsvadze N, Katsitadze G, Imnadze P, et al. Prevalence and diversity of *Bartonella* species in rodents from Georgia (Caucasus). Am J Trop Med Hyg. 2016;95(2):466-471. https://doi.org/10.4269/ajtmh.16-0041. PMID: 27162268.

93. Rozental T, Ferreira MS, Guterres A, Mares-Guia MA, Teixeira BR, Goncalves J, et al. Zoonotic pathogens in Atlantic Forest wild rodents in Brazil: *Bartonella* and *Coxiella* infections. Acta Trop. 2017;168:64-73. https://doi.org/10.1016/j.actatropica.2017.01.003. PMID: 28077317.

94. Bai Y, Kosoy MY, Lerdthusnee K, Peruski LF, Richardson JH. Prevalence and genetic heterogeneity of *Bartonella* strains cultured from rodents from 17 provinces in Thailand. Am J Trop Med Hyg. 2009;81(5):811-816. https://doi.org/10.4269/ajtmh.2009.09-0294. PMID: 19861616.

95. Gundi VA, Billeter SA, Rood MP, Kosoy MY. *Bartonella* spp. in rats and zoonoses, Los Angeles, California, USA. Emerg Infect Dis. 2012;18(4):631-633. https://doi.org/10.3201/eid1804.110816. PMID: 22469313.

96. Su Q, Chen Y, Wang B, Huang C, Han S, Yuan G, et al. Epidemiology and genetic diversity of zoonotic pathogens in urban rats (*Rattus* spp.) from a subtropical city, Guangzhou, southern China. Zoonoses Public Health. 2020;67(5):534-545. https://doi.org/10.1111/zph.12717. PMID: 32452163.

97. Kim SY, Kim TK, Kim TY, Lee HI. Geographical Distribution of *Borrelia burgdorferi* sensu lato in ticks collected from wild rodents in the Republic of Korea. Pathogens. 2020;9(11):866. https://doi.org/10.3390/pathogens9110866. PMID: 33105824.

98. Khanakah G, Kocianova E, Vyrostekova V, Rehacek J, Kundi M, Stanek G. Seasonal variations in detecting *Borrelia burgdorferi* sensu lato in rodents from north eastern

Austria. Wien Klin Wochenschr. 2006;118(23-24):754-758. https://doi.org/10.1007/s00508-006-0730-y. PMID: 17186171.

99. Kybicova K, Kurzova Z, Hulinska D. Molecular and serological evidence of *Borrelia burgdorferi* sensu lato in wild rodents in the Czech Republic. Vector Borne Zoonotic Dis. 2008;8(5):645-652. https://doi.org/10.1089/vbz.2007.0249. PMID: 18454596.

100. Barandika JF, Hurtado A, Garcia-Esteban C, Gil H, Escudero R, Barral M, et al. Tick-borne zoonotic bacteria in wild and domestic small mammals in northern Spain. Appl Environ Microbiol. 2007;73(19):6166-6171. https://doi.org/10.1128/AEM.00590-07. PMID: 17693556.

101. Moon S, Gwack J, Hwang KJ, Kwon D, Kim S, Noh Y, et al. Autochthonous lyme borreliosis in humans and ticks in Korea. Osong Public Health and Research Perspectives. 2013;4(1):52-56.

102. Hanincova K, Schafer SM, Etti S, Sewell HS, Taragelova V, Ziak D, et al. Association of *Borrelia afzelii* with rodents in Europe. Parasitology. 2003;126(Pt 1):11-20. https://doi.org/10.1017/s0031182002002548. PMID: 12613759.

103. Seo MG, Kwon OD, Kwak D. Molecular Identification of *Borrelia afzelii* from ticks parasitizing domestic and wild animals in South Korea. Microorganisms. 2020;8(5):649. https://doi.org/10.3390/microorganisms8050649. PMID: 32365723.

104. Choi YJ, Han SH, Park JM, Lee KM, Lee EM, Lee SH, et al. First molecular detection of *Borrelia afzelii* in clinical samples in Korea. Microbiol Immunol. 2007;51(12):1201-1207. https://doi.org/10.1111/j.1348-0421.2007.tb04015.x. PMID: 18094538.

105. Liu L, Baoliang X, Yingqun F, Ming L, Yu Y, Yong H, et al. *Coxiella burnetii* in rodents on Heixiazi Island at the Sino-Russian border. Am J Trop Med Hyg. 2013;88(4):770-773. https://doi.org/10.4269/ajtmh.12-0580. PMID: 23382172.

106. Mangombi-Pambou J, Granjon L, Labarrere C, Kane M, Niang Y, Fournier PE, et al. New Genotype of *Coxiella burnetii* causing epizootic Q fever outbreak in rodents, northern Senegal. Emerg Infect Dis. 2023;29(5):1078-1081. https://doi.org/10.3201/eid2905.221034. PMID: 37081672.
107. Chitanga S, Simulundu E, Simuunza MC, Changula K, Qiu Y, Kajihara M, et al. First molecular detection and genetic characterization of *Coxiella burnetii* in Zambian dogs and rodents. Parasit Vectors. 2018;11(1):40. https://doi.org/10.1186/s13071-018-2629-7. PMID: 29343277.
108. Yoo JR, Kim MS, Heo ST, Oh HJ, Oh JH, Ko SY, et al. Seroreactivity to *Coxiella burnetii* in an agricultural population and prevalence of *Coxiella burnetii* infection in ticks of a non-endemic region for Q fever in South Korea. Pathogens. 2021;10(10):1337. https://doi.org/10.3390/pathogens10101337. PMID: 34684286.
109. Truong AT, Yun BR, Lim J, Min S, Yoo MS, Yoon SS, et al. Real-time PCR biochip for on-site detection of *Coxiella burnetii* in ticks. Parasit Vectors. 2021;14(1):239. https://doi.org/10.1186/s13071-021-04744-z. PMID: 33957987.
110. Yoo JR, Heo ST, Kim M, Kim M, Kang MJ, Kim ET, et al. Coinfection of severe fever with thrombocytopenia syndrome virus and *Coxiella burnetii* in developmental stage of hard ticks in subtropical region of Korea. J Korean Med Sci. 2023;38(20):e156. https://doi.org/10.3346/jkms.2023.38.e156. PMID: 37218355.
111. Kim JH, Choi YJ, Lee KS, Kim JE, Oh JW, Moon JH. Severe fever with thrombocytopenia syndrome with Q fever coinfection in an 8-year-old girl. Pediatr Infect Dis J. 2021;40(1):e31-e34. https://doi.org/10.1097/INF.0000000000002948. PMID: 33105338.
112. Yim EK, Kim YW, Lee JS, Chang IA, Baek LJ, Song JW, et al. Detection of leptospiral DNA from field rodents by PCR. J Bacteriol Virol. 2003:177-181.

113. Dobigny G, Garba M, Tatard C, Loiseau A, Galan M, Kadaoure I, et al. Urban market gardening and rodent-borne pathogenic *Leptospira* in arid zones: a case study in niamey, Niger. PLoS Negl Trop Dis. 2015;9(10):e0004097. https://doi.org/10.1371/journal.pntd.0004097. PMID: 26437456.
114. Samir A, Soliman R, El-Hariri M, Abdel-Moein K, Hatem ME. Leptospirosis in animals and human contacts in Egypt: broad range surveillance. Rev Soc Bras Med Trop. 2015;48(3):272-277. https://doi.org/10.1590/0037-8682-0102-2015. PMID: 26108004.
115. Halliday JEB, Knobel DL, Allan KJ, de CBBM, Handel I, Agwanda B, et al. Urban leptospirosis in Africa: a cross-sectional survey of *Leptospira* infection in rodents in the Kibera urban settlement, Nairobi, Kenya. Am J Trop Med Hyg. 2013;89(6):1095-1102. https://doi.org/10.4269/ajtmh.13-0415. PMID: 24080637.
116. Izquierdo-Rodriguez E, Fernandez-Alvarez A, Martin-Carrillo N, Marchand B, Feliu C, Miquel J, et al. Pathogenic *Leptospira* species in rodents from Corsica (France). PLoS One. 2020;15(6):e0233776. https://doi.org/10.1371/journal.pone.0233776. PMID: 32502160.
117. Vitale M, Agnello S, Chetta M, Amato B, Vitale G, Bella CD, et al. Human leptospirosis cases in Palermo Italy. The role of rodents and climate. J Infect Public Health. 2018;11(2):209-214. https://doi.org/10.1016/j.jiph.2017.07.024. PMID: 28802826.
118. Cho MK, Kee SH, Song HJ, Kim KH, Song KJ, Baek LJ, et al. Infection rate of *Leptospira interrogans* in the field rodent, *Apodemus agrarius*, in Korea. Epidemiol Infect. 1998;121(3):685-690. https://doi.org/10.1017/s0950268898001691. PMID: 10030719.
119. Gu XL, Su WQ, Zhou CM, Fang LZ, Zhu K, Ma DQ, et al. SFTSV infection in rodents and their ectoparasitic chiggers. PLoS Negl Trop Dis. 2022;16(8):e0010698. https://doi.org/10.1371/journal.pntd.0010698. PMID: 36037170.

120. Rim JM, Han SW, Cho YK, Kang JG, Choi KS, Jeong H, et al. Survey of severe fever with thrombocytopenia syndrome virus in wild boar in the Republic of Korea. Ticks Tick Borne Dis. 2021;12(6):101813. https://doi.org/10.1016/j.ttbdis.2021.101813. PMID: 34411795.
121. Kang JG, Cho YK, Jo YS, Han SW, Chae JB, Park JE, et al. Severe fever with thrombocytopenia syndrome virus in ticks in the Republic of Korea. Korean J Parasitol. 2022;60(1):65-71. https://doi.org/10.3347/kjp.2022.60.1.65. PMID: 35247957.
122. Kang JG, Cho YK, Han SW, Jeon K, Choi H, Kim JH, et al. Molecular and serological investigation of severe fever with thrombocytopenia syndrome virus in cats. Vector Borne Zoonotic Dis. 2020;20(12):916-920. https://doi.org/10.1089/vbz.2020.2649. PMID: 32831006.
123. Kang JG, Cho YK, Jo YS, Chae JB, Joo YH, Park KW, et al. Severe fever with thrombocytopenia syndrome virus in dogs, South Korea. Emerg Infect Dis. 2019;25(2):376-378. https://doi.org/10.3201/eid2502.180859. PMID: 30666951.
124. Kang JG, Oh SS, Jo YS, Chae JB, Cho YK, Chae JS. Molecular detection of severe fever with thrombocytopenia syndrome virus in Korean domesticated pigs. Vector Borne Zoonotic Dis. 2018;18(8):450-452. https://doi.org/10.1089/vbz.2018.2310. PMID: 29893622.
125. Kang JG, Cho YK, Jo YS, Chae JB, Oh SS, Kim KH, et al. Prevalence of severe fever with thrombocytopenia syndrome virus in black goats (*Capra hircus coreanae*) in the Republic of Korea. Ticks Tick Borne Dis. 2018;9(5):1153-1157. https://doi.org/10.1016/j.ttbdis.2018.04.018. PMID: 29724620.
126. Oh SS, Chae JB, Kang JG, Kim HC, Chong ST, Shin JH, et al. Detection of severe fever with thrombocytopenia syndrome virus from wild animals and *Ixodidae* ticks in the

Republic of Korea. Vector Borne Zoonotic Dis. 2016;16(6):408-414. https://doi.org/10.1089/vbz.2015.1848. PMID: 27043361.

127. Hwang J, Kang JG, Oh SS, Chae JB, Cho YK, Cho YS, et al. Molecular detection of severe fever with thrombocytopenia syndrome virus (SFTSV) in feral cats from Seoul, Korea. Ticks Tick Borne Dis. 2017;8(1):9-12. https://doi.org/10.1016/j.ttbdis.2016.08.005. PMID: 27542506.

128. Tsuru M, Suzuki T, Murakami T, Matsui K, Maeda Y, Yoshikawa T, et al. Pathological characteristics of a patient with severe fever with thrombocytopenia syndrome (SFTS) infected with SFTS Virus through a sick cat's bite. Viruses. 2021;13(2):204. https://doi.org/10.3390/v13020204. PMID: 33572914.

129. Kida K, Matsuoka Y, Shimoda T, Matsuoka H, Yamada H, Saito T, et al. A case of cat-to-human transmission of severe fever with thrombocytopenia syndrome virus. Jpn J Infect Dis. 2019;72(5):356-358. https://doi.org/10.7883/yoken.JJID.2018.526. PMID: 31366857.

130. Seo JW, Kim D, Yun N, Kim DM. Clinical update of severe fever with thrombocytopenia syndrome. Viruses. 2021;13(7):1213. https://doi.org/10.3390/v13071213. PMID: 34201811.

131. Yun SM, Park SJ, Kim YI, Park SW, Yu MA, Kwon HI, et al. Genetic and pathogenic diversity of severe fever with thrombocytopenia syndrome virus (SFTSV) in South Korea. JCI Insight. 2020;5(2):e129531. https://doi.org/10.1172/jci.insight.129531. PMID: 31877113.

Figure legends

Fig 1. Maps showing the regions where rodent-borne pathogens were detected in the Republic of Korea. Marks are differently indicated according to each pathogen.

Fig 2. Phylogenetic tree inferred by maximum-likelihood analysis using the K2 + G model of 16S rRNA gene sequence of *Anaplasma phagocytophilum*. The numbers at the nodes are bootstrap values expressed as percentage of 1000 replicates. Scale bar indicates nucleotide substitution per site. Samples sequenced from *Apodemus agrarius* are shown in filled circles.

Fig 3. Phylogenetic analysis based on *ITS* region of *Bartonella* spp. (maximum-likelihood analysis using the Tamura 3-parameter + G + I model with of 1000 replicates). Scale bar indicates nucleotide substitution per site. Sequences determined from *Apodemus agrarius* are indicated in filled circles.

Fig 4. Maximum-likelihood phylogenetic tree using the Tamura-Nei model based on *ospA* gene of *Borrelia* spp. Bootstrap values were calculated with 1000 replicates of the alignment. Scale bar indicates nucleotide substitution per site. Sequences obtained from *Apodemus agrarius* are symbolized in filled circles.

Fig 5. Maximum-likelihood phylogenetic tree from *IS1111* gene of *Coxiella burnetii*. The evolutionary analysis was inferred using the Kimura 2-parameter model. Bootstrap values (1000 replicates) are indicated in each node. Scale bar implies nucleotide substitution per site. Sequences determined from *Apodemus agrarius* are highlighted in filled circles.

Fig 6. Phylogenetic analysis based on *rpoB* gene of *Leptospira interrogans*. The tree was inferred in MEGA X using maximum-likelihood and Kimura 2-parameter with 1000 replicates. Scale bar implies nucleotide substitution per site. Sequences obtained from *Apodemus agrarius* are shown in filled circles.

Fig 7. Phylogenetic tree of the severe fever with thrombocytopenia syndrome virus based on

the analysis of partial sequences of small segments. Maximum-likelihood analysis was used to construct by the Kimura 2-parameter model (1000 bootstrap replicates). Scale bar implies nucleotide substitution per site. The sequences identified from *Apodemus agrarius* are indicated in filled circles.

Figure 1

Click here to access/download;Figure;Fig. 1.jpg

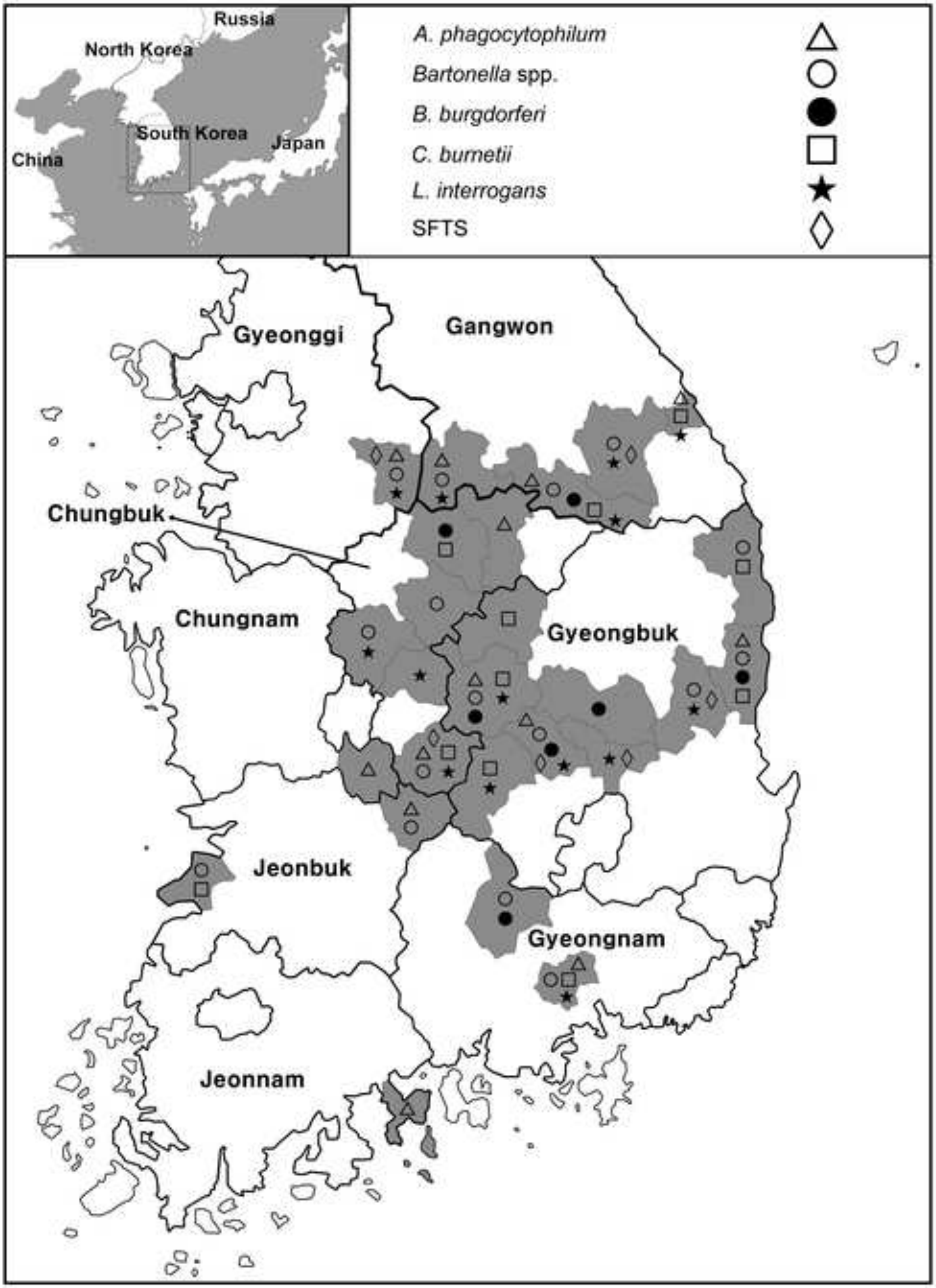

Figure 2

Click here to access/download;Figure;Fig. 2.pptx

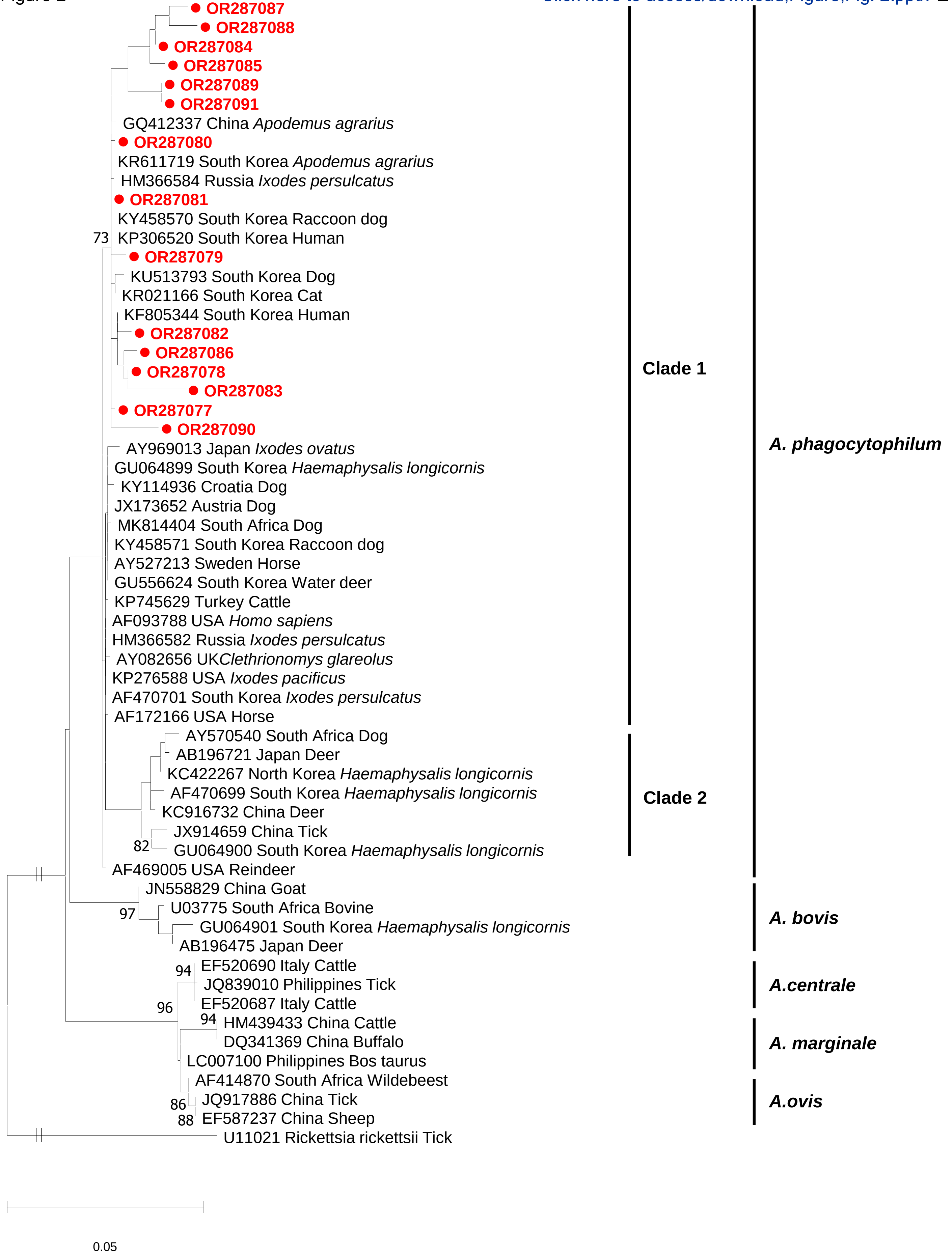

Figure 3

Click here to access/download;Figure;Fig. 3.pptx

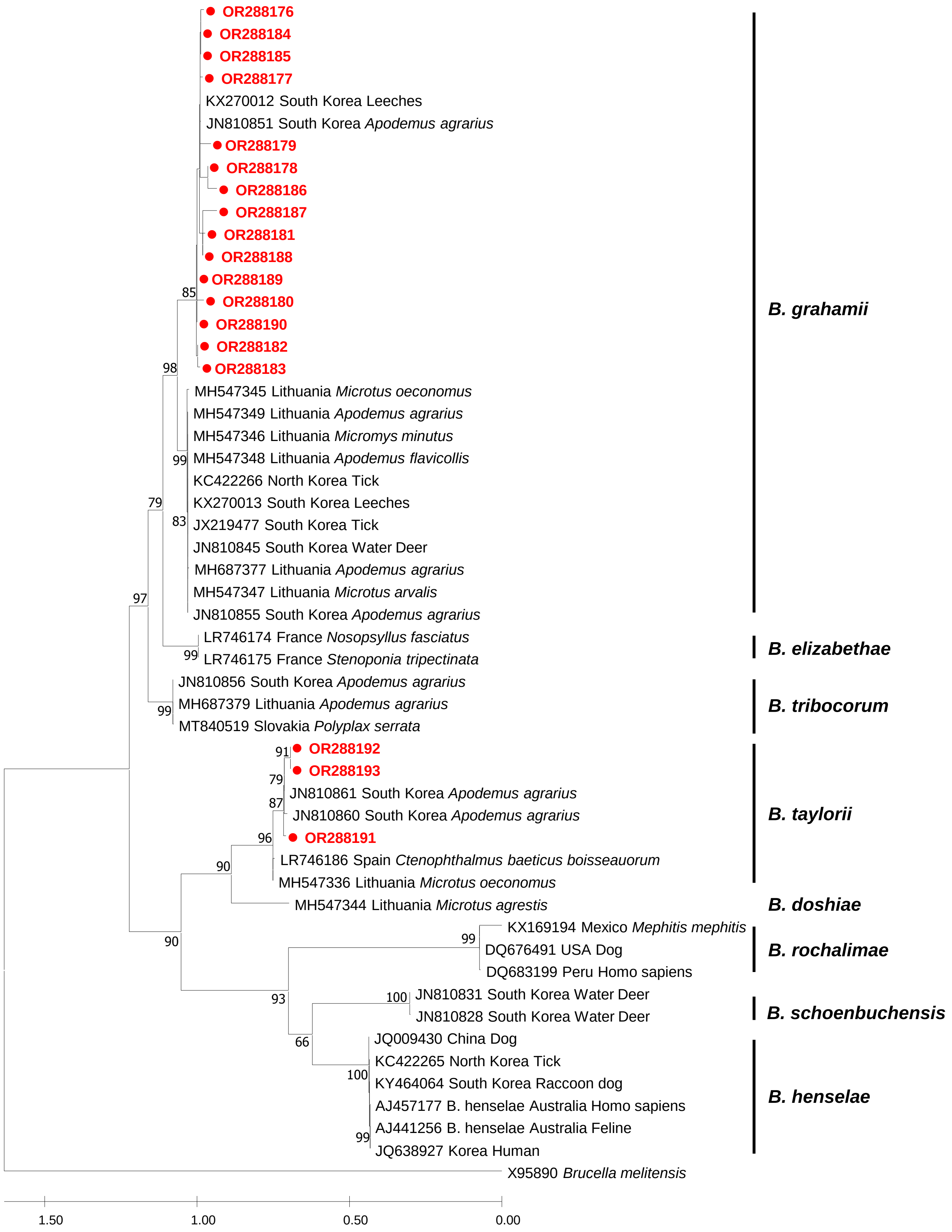

Figure 4 Click here to access/download;Figure;Fig. 4.pptx

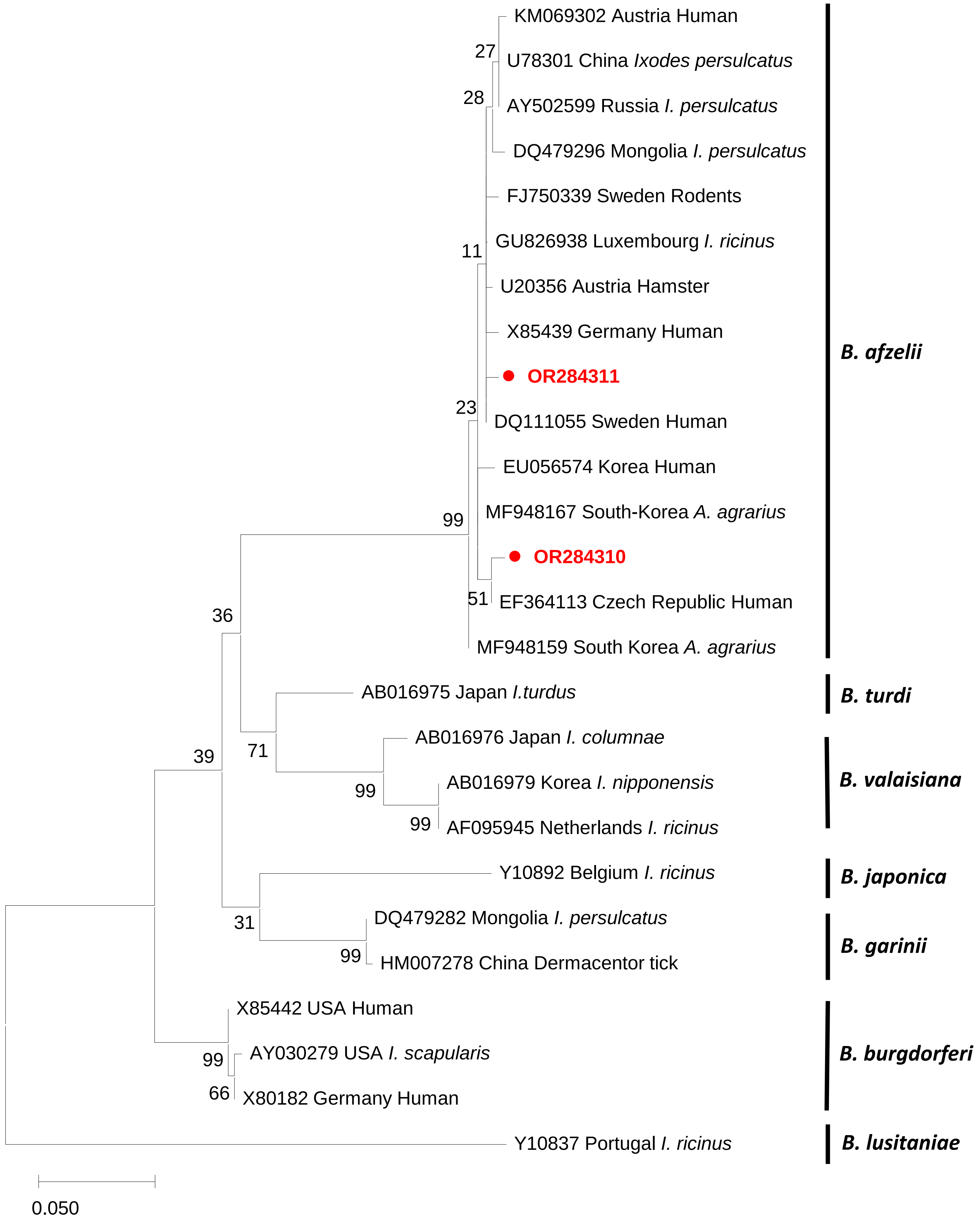

Figure 5

Click here to access/download;Figure;Fig. 5.pptx

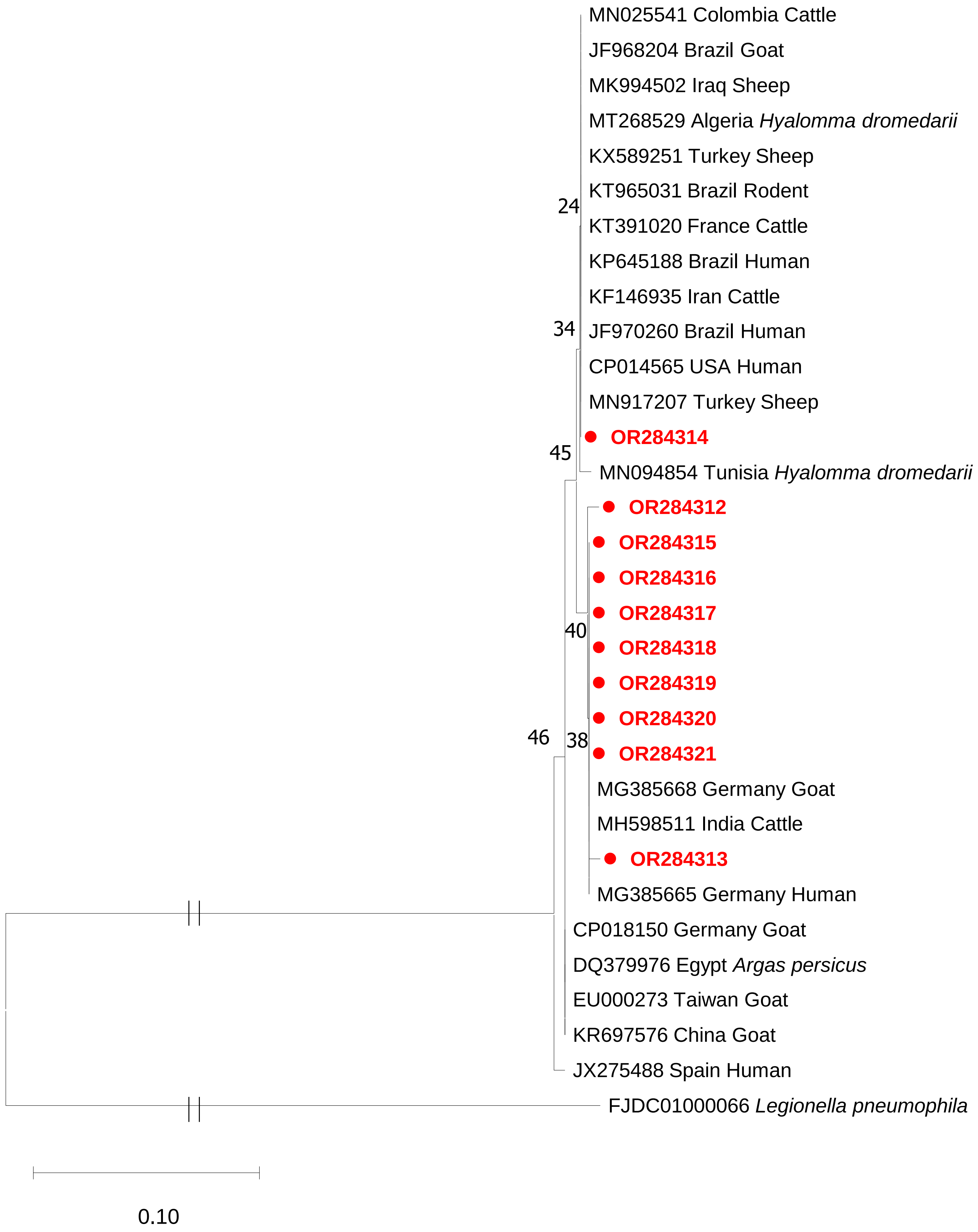

Figure 6

Click here to access/download;Figure;Fig. 6.pptx

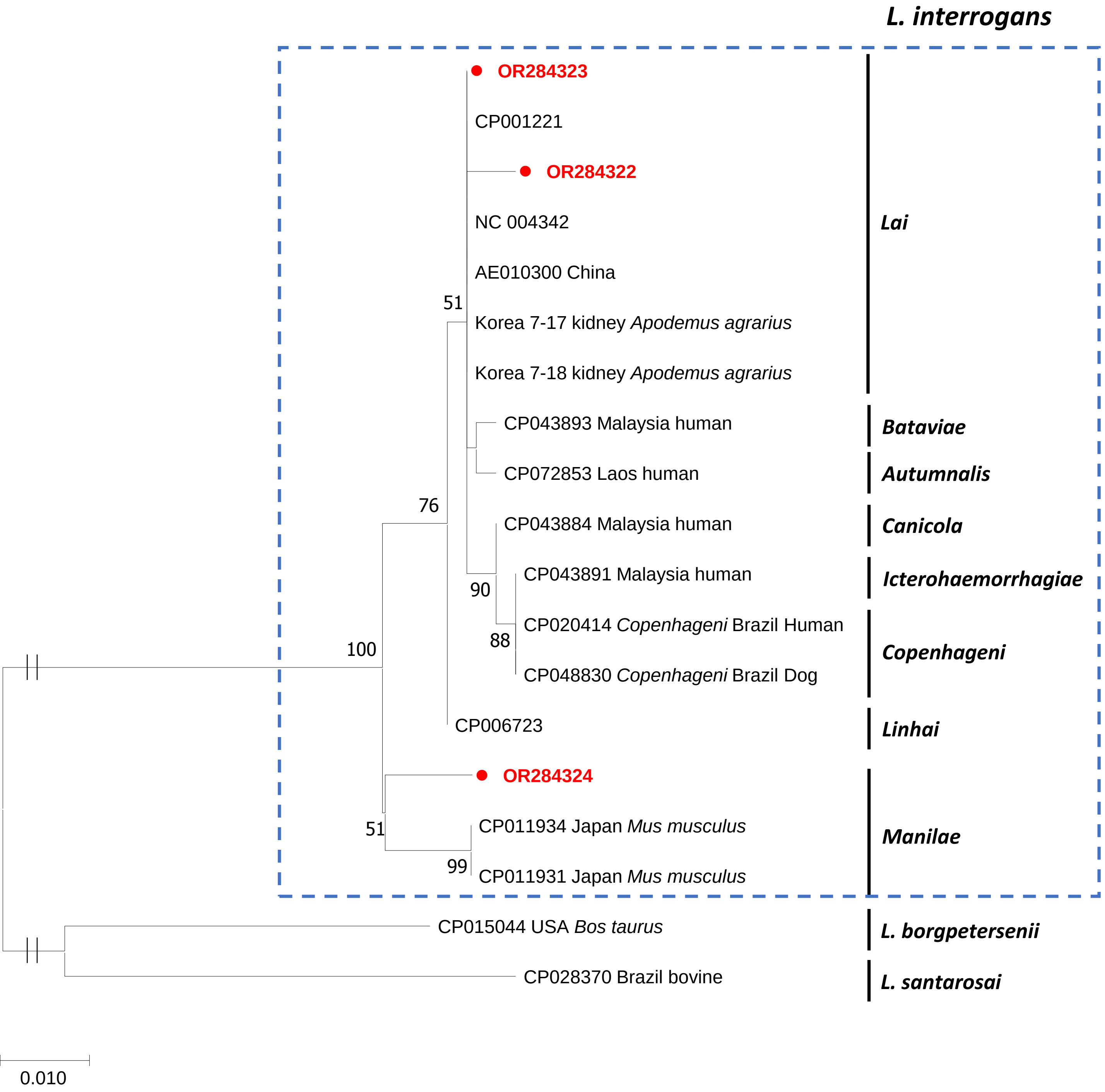

Figure 7

Click here to access/download;Figure;Fig. 7.pptx

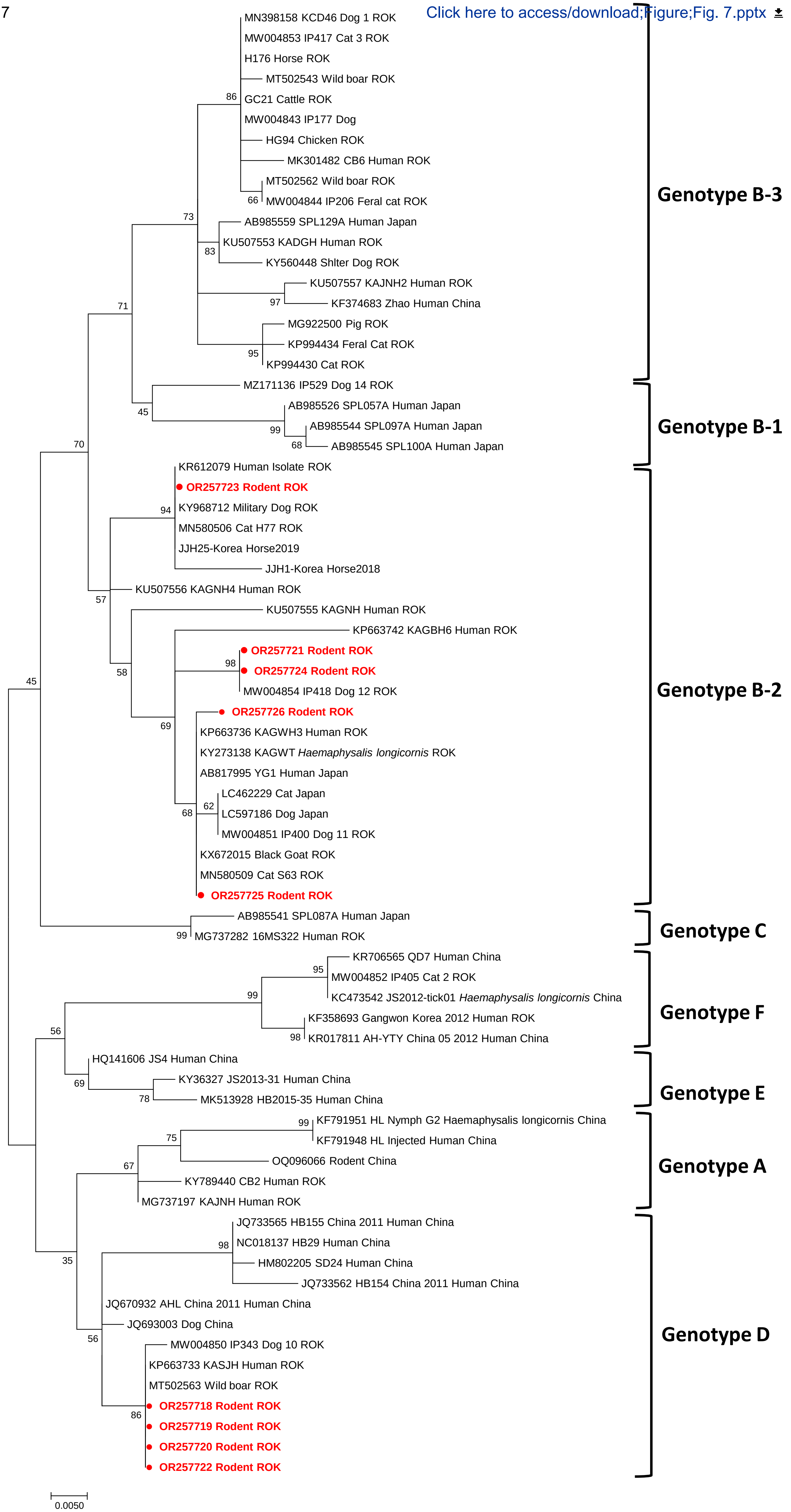

Table Click here to access/download;Table;Tables.docx

Table 1. Primer information used for PCR analysis.

Pathogens	Target genes	Sequences (5′–3′)	Sizes (bp)	Annealing temp./Time	References
Anaplasma phagocytophilum	16S rRNA	TCCTGGCTCAGAACGAACGCTGGCGGC	1433	50°C/30 s	Han et al., 2019
		AGTCACTGACCCAACCTTAAATGGCTG			
		GTCGAACGGATTATTTTTATAGCTTGC	926	56°C/30 s	
		CCCTTCCGTTAAGAAGGATCTAATCTCC			
Bartonella spp.	*ITS*	TTCAGATGATGATCCCAAGC	639	55°C/30 s	Ko S 2016
		AACATGTCTGAATATATCTTC			
		CCGGAGGGCTTGTAGCTCAG	499	55°C/30 s	
		CACAATTTCAATAGAAC			
Borrelia spp.	*ospA*	GGGAATAGGTCTAATATTAGCC	665	42°C/60 s	Priem S 1998
		CACTAATTGTTAAAGTGGAAGT			
		GCAAAATGTTAGCAGCCTTGAT	392	56°C/60 s	
		CTGTGTATTCAAGTCTGGC			
Coxiella burnetii	*IS1111*	TATGTATCCACCGTAGCCAGTC	687	54°C/30 s	Parisi A 2006
		CCCAACAACAACCTCCTTATTC			
		GAGCGAACCATTGGTATCG	203	54°C/30 s	
		CTTTAACAGCGCTTGAACGT			
Leptospira interrogans	*rpoB*	GTTCCAACATGCAACGYCAR	1649	52°C/60 s	Bang MS 2019
		GTTGAAGGATTCRGGRATAC			
		TYATGCCKTGGGAAGGWTAC	1023	56°C/30 s	
		GCATRTCRTCKGACTTGATG			
SFTSV	S	CATCATTGTCTTTGCCCTGA	461	52°C/40 s	Yoshikawa T et al 2014
		AGAAGACAGAGTTCACAGCA			
		AAYAAGATCGTCAAGGCATCA	346	55°C/40 s	Oh SS 2015
		TAGTCTTGGTGAAGGCAT CTT			

*SFTS: severe fever with thrombocytopenia syndrome virus

Table 2. Number of rodents captured by regions.

Province/Species	*Apodemus agrarius*	*Rattus norvegicus*	Unknown	Total
Gyeonggi	12	–	2	14
Gangwon	18	–	5	23
Chungbuk	19	–	3	22
Chungnam /Daejeon	4	–	–	4
Jeonbuk	13	–	–	13
Jeonnam	4	–	–	4
Gyeongbuk	76	4	9	89
Gyeongnam	5	1	–	6
Total	151	5	19	175

"–": none of rodents captured

Table 3. Number of positive samples in which pathogens were identified from captured rodents.

Variables	*A. phagocytophilum*	*Bartonella* spp.	*Borrelia* spp.	*C. burnetii*	*L. interrogans*	SFTSV
**Species**						
Apodemus agrarius (*n* = 151)	21	72	5	22	23	9
Rattus norvegicus (*n* = 5)	–	1	–	3	1	–
Total (*n* = 156)	21	73	5	25	24	9
**Province**						
Gyeonggi (*n* = 12)	1	5	–	–	2	2
Gangwon (*n* = 18	4	12	1	5	6	1
Chungbuk (*n* = 19)	3	10	1	2	6	2
Chungnam/Daejeon (*n* = 4)	1	–	–	–	–	–
Jeonbuk (*n* = 13)	3	8	–	3	–	–
Jeonnam (*n* = 4)	1	–	–	–	–	–
Gyeongbuk (*n* = 80)	7	32	2	14	6	4
Gyeongnam (*n* = 6)	1	6	1	1	4	–
Total (*n* = 156)	21 (13.5%)	73 (46.8%)	5 (3.2%)	25 (16.0%)	24 (15.4%)	9 (5.8%)

Table 4. Co-infections of two or three pathogens detected from captured rodents.

Pathogens	No. of positive samples
A. phagocytophilum + *Bartonella* spp.	7
A. phagocytophilum + *Borrelia* spp.	1
A. phagocytophilum + *C. burnetii*	1
Bartonella spp. + *Borrelia* spp.	2
Bartonella spp. + *C. burnetii*	7
Bartonella spp. + *L. interrogans*	10
Bartonella spp. + SFTSV	3
C. burnetii + *L. interrogans*	2
A. phagocytophilum + *Bartonella* spp.+ *C. burnetii*	1
A. phagocytophilum + *Bartonella* spp.+ *L. interrogans*	3
A. phagocytophilum + *Bartonella* spp.+ SFTSV	2
A. phagocytophilum + *C. burnetii* + *L. interrogans*	1
Bartonella spp. + *Borrelia* spp. + *C. burnetii*	1
Bartonella spp. + *C. burnetii* + *L. interrogans*	1
Bartonella spp. + *L. interrogans* + SFTSV	2
