# Supplementary material for: Molecular surveillance of zoonotic pathogens from wild rodents in the Republic of Korea
Source: PLoS Negl Trop Dis. 2024 Jul 8;18(7):e0012306. doi: 10.1371/journal.pntd.0012306 (PMC11257403; doi:10.1371/journal.pntd.0012306)
Supplement: Attachment — Submitted filename: Response to reviewers comments.docx [file pntd.0012306.s004.docx]

**We thank the reviewers for their careful reading and critical insights. These constructive comments were valuable and helpful for revising and improving our manuscript. We have provided detailed responses to the points raised by the reviewers and have modified our manuscript accordingly. The revised portions have been highlighted in red in the revised manuscript**. **We hope that the** **revised manuscript will be suitable for publication in PLoS Neglected Tropical Diseases.**

<b>Methods
Reviewer #1: There is some basic information that should be included in the methods.

1. the general description of “near rivers, valleys, farms, mountains, and lakes” is ambiguous. Were any other data collected (specific habitats, environmental conditions, etc)? Perhaps the authors could use more helpful descriptors (e.g., periurban? rural? near urban centers?) This would help their discussion, where they conclude that “rodents were trapped from areas with frequent movement of people, which may be associated with a high probability of disease transmission…” They state that the sites were selected by SFTS cases, but should provide more information to help understand what/where exactly they sampled, ecologically.

**Response: We agree with this comment and have provided information as the reviewer’s suggestion. Please see lines 170-173.**

2. The authors must state how the rodents were identified. Defining how the rodents were identified becomes an issue as ~11% of the captured rodents were “unknown” species. Were the authors simply more confident in their identification of (the very distinctive) Apodemus agrarius and Rattus norvegicus, but were less confident to identify other rodents? If they identified A. agrarius as “rodent with a stripe” then they should state that as the identification method - how did they know it was not a Sicista sp? How did they discriminate between Rattus rattus and Rattus norvegicus?

**Response: We understand the reviewer’s concern. Because** ***Apodemus agrarius* species have very distinct characteristics, namely black stripe on their back, it’s easy to discriminate from others. In case of *Rattus* spp., we distinguished *Rattus rattus* (black rat) and *Rattus norvegicus* (brown rat) by morphological characteristics, especially color. In this study, the rodent species were morphologically identified. Please see line 177. Moreover, one reviewer suggested to remove the results of *Rattus norvegicus*, and we removed all.**

3. The authors have to be more specific about their geographic scale. They refer to “province” in Tables 2 and 3, but display subdivisions of provinces in Figure 1. In the text they refer to “regions” and it seems region = province, but it is not clear. This should be very clearly defined, specifically stating the scale at which the data were analyzed, and explaining why the map shows a different scale than how the data were analyzed. For example, there appear to be 9 subdivisions of Gyeongbuk for which presence/absence is noted – are these the “regions” referred to in the text? Are these all regions that were sampled? Other examples: L276 – “Four regions” but 7 points are shown on Figure 1; L286 – “Five different regions” but 11 points are shown on Figure 1; L309 – “four different regions” but 6 points are shown on Figure 1; L330 – “found in all regions”.

**Response: We’re sorry for the confusion and made a mistake. A province is larger than a region. We replaced all region with province in the manuscript. Please see lines 167-168, 257, 268, 284, 299, 310, 333, 363, and 364. To provide more accurate information, we have also added supplementary table 1.**

4. One has to consider trapping effort for “prevalence” to be meaningful. The authors wish to make conclusions comparing the observed infection ratios between pathogens/regions/species and make comparisons to historical data (e.g., L226-L231, L232-L235 and Table 3). The probability of detecting something increases with increased sampling. For example, I would argue that there is very little that they can conclude about the vector/reservoir status of R. norvegicus based on 5 captures. So, in addition to the above mentioned points (describing the sampling sites and clarifying the geographic scale), the authors must state and/or consider the sampling effort (trap-nights per sampling unit, e.g., per site? region? province?) when analyzing their data. Otherwise they must limit their presentation, analysis, and discussion to presence/absence in the ROK.

**Response: We agree with this comment. At every spot (region), a total of 60 traps were installed, but the number of captured rodents was different in each spot (region). We have provided this and please see line 174. Moreover, we removed the data from *R. norvegicus* because one reviewer suggested to remove it.**

5. If the authors define their sampling strategy and state their sampling effort with respect to geographic scale, they have to include correct statistical comparisons to make any inferences. For example, L259 – “The most” is misleading. Even though Bartonella were detected in 48% of A. agrarius, the confidence interval for the estimated number of R. norvegicus expected to be Bartonella-positive is ~1-70% based on 1/5 captures, i.e., there is no difference in infection ratio.

**Response: We agree with this comment and understand the reviewer’s concern. One reviewer suggested to remove the results of *Rattus norvegicus*, and we have replaced “the most” with “frequently”. Please see line 253. Also, we have provided 95% CI for each pathogen result. Please see lines 253-256, and 261.**

Reviewer #2: -Are the objectives of the study clearly articulated with a clear testable hypothesis stated? Yes

Reviewer comments were added to uploaded document.

**Response: We have provided all in a separate page.**

Reviewer #3: - **Objectives and Hypothesis**: The objectives of the study are outlined but could be more explicitly connected to a clear, testable hypothesis. Clarifying the hypothesis would enhance the study's focus and provide a clearer framework for evaluation.
- **Study Design**: The design, involving molecular surveillance across various regions, appears appropriate for addressing the broad objectives related to pathogen prevalence. However, the justification for specific methodological choices (e.g., nested PCR) need to be strengthened.
- **Population Description**: The rodent population is clearly described, and the choice of species seems appropriate for the study's focus on zoonotic pathogens. The geographical and ecological breadth covered is a strength. Although the sample size is substantial for a surveillance study of this nature, a power analysis to justify the sample size in relation to expected pathogen prevalence is missing and would enhance the methodology.
- **Statistical Analysis**: There is minimal discussion on the statistical methods used for analyzing the data. Expanding on this, especially regarding the handling of co-infections and multiple comparisons, would support the conclusions drawn.

**Response: We agree with this comment, and to detect all pathogens, we used the nest PCR methods. Moreover, statistical analysis was performed as the reviewer’s suggestion. According to our results, there was no statistically significant differences between co-infection and multiple infections. We have provided the details. Please see lines 228-233, 249-252, and 352-354.**

<b>Results
Reviewer #1: Table 3 – I suggest removing R. norvegicus from this table entirely. The status of the five specimens can be easily stated in the text. I think it is important to know which of the detections per province were from each species, and with 151 individuals, a Table is the best way to display the data for A. agrarius, but the R. norvegicus data makes these numbers ambiguous.

**Response: We agree with this comment and have deleted all data from *R. norvegicus*. Please see Table 4.**

Reviewer #2: -Does the analysis presented match the analysis plan? Yes
-Are the results clearly and completely presented? No
-Are the figures (Tables, Images) of sufficient quality for clarity? Yes

Reviewer comments were added to uploaded document.

**Response: We have provided all in a separate page.**

Reviewer #3: - **Clarity and Completeness**: The results are presented with significant findings regarding pathogen prevalence and co-infections. However, detailing in which tissues each pathogen was detected would provide a significant improvement.
- **Quality of Figures and Tables**: The provided figures and tables are adequate but could be enhanced for clarity, particularly regarding the visualization of co-infection patterns and their distribution across sampled regions.

**Response: Regarding those, we mentioned in materials and methods. Please see lines 185-187 and 193-197. We have revised as the reviewer’s suggestion. Please see new figure 1.**

<b>Conclusions

Reviewer #1: The authors make conclusions concerning relative prevalence/occurrence of the rodents and pathogens with respect to geography. Without a more rigorous sampling strategy, or at least a better described/defined sampling scheme, they are limited in making some of these comparisons. In addition, the discussion includes details that are not completely relevant to the study design (as I have understood it). They should focus on presence/prevalence. They have no data on pathogenicity, so I recommend removing these points. I have included an edited pdf copy of the manuscript with my suggestions of things to delete for their consideration, and they could probably remove more.

**Response: We agree with this comment and have provided the sampling method (lines 170-174). In addition, we revised as the reviewer’s suggestion.**

The study has an additional limitation that should absolutely be addressed. The authors chose to use PCR or conventional RT-PCR for identification, yet failed to generate sequences from a proportion of the “positives”. The authors should address this specifically in the discussion: is this an acceptable ratio in similar studies? Should these be considered “putative” positive? Why weren’t more sensitive and specific methods used (e.g., RTqPCR)? Could this also influence the ratio when comparing to other, previously published molecular surveys (e.g., Kim et al. 2022 Parasite & Vectors 15:486, doi: 10.1186/s13071-022-05608-w)?

**Response: We agree with this comment and understand the reviewer’s concern. At that time, we didn’t have real-time PCR machine so that’s the reason why we used the nested PCR. Please see lines 209-211, 369-379, 431-433, and 483-485.**

Another minor limitation that the authors should address is that they provide no information about the structure of the cohort (males/females, adult/juvenile, etc). This is not completely necessary to include in the analysis, but should be mentioned/considered in the discussion as it may affect the observed prevalence (infection ratio); and I suspect it had a very large affect on the trapping success.

**Response: We agree with this comment. Unfortunately, we could not provide information regarding adult/juvenile. As the reviewer’s suggestion, we analyzed the data of male and female and provided the results about that. Please see lines 248-252, 403-405, and Table 3.**

Reviewer #2: -Are the conclusions supported by the data presented? Yes
-Are the limitations of analysis clearly described? Yes
-Do the authors discuss how these data can be helpful to advance our understanding of the topic under study? Yes
-Is public health relevance addressed? Yes

Reviewer comments were added to uploaded document.

**Response: We have provided all in a separate page.**


Reviewer #3: - **Support by Data**: The conclusions drawn about the prevalence and public health significance of the detected pathogens are generally supported by the data, albeit with the caveat that the methodological limitations could impact these findings.
- **Limitations**: The discussion of limitations, especially regarding methodological choices (nested PCR, partial genome sequencing), is needed. Acknowledging these could strengthen the study's credibility.

**Response: We agree with this comment and provided them. Please see lines 209-211, 369-379, 431-433, and 483-485.**

- **Advancement of Understanding**: The manuscript makes a valuable contribution by mapping zoonotic pathogen prevalence in rodents in the ROK.


<b>Summary and General Comments</b></br></br>
Use this section to provide overall comments, discuss strengths/weaknesses of the study, novelty, significance, general execution and scholarship. You may also include additional comments for the author, including concerns about dual publication, research ethics, or publication ethics. If requesting major revision, please articulate the new experiments that are needed.

Reviewer #1: The authors screen for the presence of some bacterial pathogens and one arbovirus in 2 rodent species captured at targeted sites, based on human cases of the arbovirus. The main goal was to characterize the presence of these zoonotic pathogens in the Republic of Korea in two rodent species. They screen by molecular diagnostic methods, and report the infection ratios by species and by geographic unit. They also included some phylogenetic analyses of the detected pathogens, including some (but not all) of the "positives". Overall the methods require a bit more clarification, because I think the manuscript needs a better perspective on what can/not be inferred from the study design and data. As explained in other comments above, the geographic unit and sampling effort need to be clearly defined if the authors wish to infer prevalence based on geography. The phylogenetic analysis is fine, but the authors should limit the conclusions they can make from these data
concerning pathogenicity. Simply stated, this could be a simple epidemiological survey for multiple pathogens. However, the authors could make some additional inferences (e.g., geographic distribution) if they are more scientific about the approach. Therefore, the discussion needs major revision, and that will depend on if/how the authors improve the methods.

**Response: We agree with this comments and have revised as the reviewer’s suggestion.**

In addition to the major points mentioned above, here are some minor suggestions / clarifications that would help the mansucript:
L135-L136: SFTSV was only briefly Huaiyangshan banyangvirus, has been Dabie bandavirus since 2018, and is now Bandavirus dabieense according to ICTV

**Response: We agree with this comments and have changed it. Please see line 136 and 137.**

L147 – I would rephrase this as “…rodents may be involved in the transmission cycles of various diseases.” Particularly as this is the hypothesis they intend to investigate.

**Response: We have modified this and please see lines 147-148.**

L153 “…to investigate the occurrence of some rodent-borne diseases…”

**Response: We agree with this comments and have changed it. Please see lines 154-155.**

L222 – I am confused by this sentence. I was under the impression that the authors only captured rodents in the ROK, but here they state that A. agrarius was captured “mostly” in ROK (and also somewhere else?). Perhaps the authors mean that A. agrarius was found at all sites (?) but R. norvegicus was only captured at 2 sites? Two regions?

**Response: We are sorry for the confusion and have modified them. Please see lines 240-241.**

L172 – define “serum separating tube (SST)”

**Response: We have changed it. Please see line 179.**

L267 – “…formed the same group as B. grahamii found in leeches (KX270012) and found in A. agrarius (JN810851) reported in the ROK…”

**Response: We agree with this comments and have revised them. Please see lines 289-290.**

L279 and Figure 1 – B. burgdorferi is listed in the figure but the phylogenetic analysis in Figure 4 and text L279 state that this is B. afzelii. Please clarify in the text or write “B. burgdorferi s.l.” on the figure.

**Response: We agree with this comments and have changed figure 1. Please see new figure 1.**

L292-293 what was the level of bootstrap support required to delineate a “clade”

**Response: Even though 1,000 replicates of bootstrap were used to evaluate the reliability, the bootstrap values in each node were low. We understand the reviewer’s concern. Nevertheless, there are several clades within the phylogenetic tree.**

L322 “prevalence” – perhaps “infection ratio” is better.

**Response: We have changed it. Please see line 345.**

L324 “most common species” in the ROK?

**Response: In this study, only *A. agrarius* was used, and this sentence was deleted as it is no longer needed.**

L335-L337 – no. The results demonstrate that the rodents have contact with some select pathogens. However, I agree with the next sentence, “Moreover, these data provide valuable information for evaluating the potential risk of rodents in public health.” The authors do not provide this analysis, so they should limit their conclusions concerning transmission risk – and should especially limit or qualify their inferences regarding prevalence unless they can provide more methodological data. The data are indeed valuable, but the analysis/interpretation should be improved.

**Response: We have deleted them. Moreover, the discussion section has been improved.**

L356-L358 – please cite evidence of the burden of H. longicornis on A. agrarius, and the role in A. phagocytophilum transmission/maintenance.

**Response: We agree with this comments and have provided cites. Please see line 393.**

L367 – The authors should discuss other possible influences to address potential limitations in their study design. Different seasons, different years, different climates, different habitats, different age structure, etc. I think these topics would apply to any of the pathogens included here, as technically A. agrarius has a rather large distribution…

**Response: We only compared the different sex and provinces and provided them in the results. Please see lines 354-362, 403-405, and 418-421.**

L379 – “…is the predominant species found in rodents”

**Response: We have revised it as the reviewer’s suggestion. Please see line 418.**

L384 – “sequence diversity”.

**Response: One reviewer suggested to remove these sentences.**

**We thank the reviewers for their careful reading and critical insights. These constructive comments were valuable and helpful for revising and improving our manuscript. We have provided detailed responses to the points raised by the reviewers and have modified our manuscript accordingly. The revised portions have been highlighted in red in the revised manuscript**. **We hope that the** **revised manuscript will be suitable for publication in PLoS Neglected Tropical Diseases.**

**Reviewer Comments**

**Minor**

Line 48 – The statement that rodents live anywhere in the world is incorrect.

**Response:** **We have changed. Please see line 49.**

Line 65 – Add other transmission routes through which indirect transmission of zoonotic diseases from rodents occur.

**Response:** **We have added as the reviewer’s suggestion. Please see line 66.**

Line 127 – Climate change is not a qualifier for heavy rainfall and flooding to occur.

**Response:** **We’re sorry for the confusion and have modified it. Please see lines 128-129.**

Line 166 – It would be informative to know how long the capture duration was a particular site.

**Response:** **We have provided as the reviewer’s suggestion. Please see lines 175-176.**

Line 169 – How were captured rodents identified and were identification of rodents confirmed through molecular identification methods?

**Response:** **Captured rodents were morphologically identified, and molecular analysis was not performed**. **We have added how the rodents were identified.** **Please see line 177. Moreover, one reviewer suggested to remove the data from *Rattus norvegicus,* so we deleted all.**

Line 172 – Write out abbreviation of SST.

**Response:** **We have provided as the reviewer’s suggestion. Please see line 179.**

Line 203 – State reference sequence accession numbers used to determine similarity.

**Response:** **We agree with this comment. Because many reference sequences were used for each pathogen, we were not able to add all accession numbers to the manuscript and instead, we have provided all references in a phylogenetic tree.**

Line 203 - State which tool was used to determine sequence similarity.

**Response:** **We used Geneious Prime software to determine the sequence similarity and have provided as the reviewer’s comment. Please see line 215.**

Line 217 – It would be informative to know if information about the rodents such as age and sex was collected during capturing of rodents.

**Response:** **We were not able to provide the age of captured rodents, but we have added information of sex. Please see lines 238-239.**

Line 239 – Sentence not appropriate in Results should be moved to Discussion section.

**Response:** **We agree with this comment and removed these sentences.**

Table 3 – Missing description of the hyphen.

**Response:** **We have provided the description of the hyphen. Please see Table 4.**

**Major**

I suggest the manuscript undergoes major revisions to improve the writing to address sentence construction and grammar, below is a limited list of examples of concern.
Line 49 – Sentence construction and word choice.

**Response:** **The manuscript has been carefully reviewed by an experienced editor whose first language is English.** **We have revised the sentence. Please see lines 50-51.**

Line 220 – Sentence construction and word choice.

**Response:** **We have revised the sentence. Please see lines 239-240.**

Line 228 – Sentence not needed.

**Response: We have deleted it as the reviewer’s suggestion.**

Line 231 - Sentence construction and word choice.

**Response: We have revised the sentence. Please see lines 256-257.**

Line 238 – SFTSV wasn’t co-infected with *Bartonella* spp. rather the rodent was co-infected with SFTSV and *Bartonella* spp.

**Response: We have deleted this sentence because similar sentences were in results.**

Line 204 – How were sequences of different length treated and used for sequence alignment and phylogenetic analysis in relationship to study obtained and reference sequences?

**Response: We understand the reviewer’s concern. For the accuracy of all results, we attempted to use a length that was approximately similar to the reference sequence. If reference sequences were too long, they were trimmed based on our sequences and used for analysis.**

Figures 2, 3, 4, 5, 6, and 7 – The rodent species and the province of where the sample was collected needs to be added to the accession number.

**Response: We agree with this comment and have modified all figures as the reviewer’s suggestion. We have provided them with region and province. Moreover, one reviewer suggested to remove *Rattus norvegicus*, and we included only *Apodemus agrarius* in all figures.** **Please see all revised figures.**

Figure 2, 3, 6 – Bootstrap values are missing from smaller branches.

**Response: We have provided all bootstrap values on missing branches as the reviewer’s suggestion.**

Figure 7 – Sample names aren’t uniform to what was used in other figures containing phylogenetic trees.

**Response: We agree with this comment and have revised all. Please see all figures.**

Table 2 - Since no analysis were performed on unknown samples and they aren’t discussed in manuscript it be removed from the table.

**Response: We agree with this comment and have removed information of unknown samples. Please see Table 2.**
