# Supplementary material for: Molecular surveillance of zoonotic pathogens from wild rodents in the Republic of Korea
Source: PLoS Negl Trop Dis. 2024 Jul 8;18(7):e0012306. doi: 10.1371/journal.pntd.0012306 (PMC11257403; doi:10.1371/journal.pntd.0012306)
Supplement: Attachment — Submitted filename: Response to reviewers comments.docx [file pntd.0012306.s005.docx]

**We thank the reviewers for their careful reading and critical insights. These constructive comments were valuable and helpful for revising and improving our manuscript. We have provided detailed responses to the points raised by the reviewers and have modified our manuscript accordingly. The revised portions have been highlighted in red in the revised manuscript**. **We hope that the** **revised manuscript will be suitable for publication in PLoS Neglected Tropical Diseases.**

Reviewer #1: The authors have improved the methods description by now including a table with regions sampled within provinces, as well as defining ecological categories that describe each region. They added more information, but there the sampling effort is still ambiguous. The authors must be very clear about trap effort. Per the methods, they define 1 trap-night = 60 traps. *State how many trap-nights were performed in each region.*

**Response: We agree with this comment and have provided information as the reviewer’s suggestion. To capture rodents, 60 traps per night were set in each region. Please see lines 175-176.**

Reviewer #2: Line 173-178 – Please clarify, was each capture location only sampled once or were they sampled multiple times?

**Response: We agree with this comment. To capture rodents, 60 traps per night were set in each region and each capture location was sampled only once. Please see lines 176-177.**

Reviewer #1: L316 (previously L293) The authors have included bootstrap values on the phylogenetic trees. However, they did not answer my previous question concerning the use of the word "clades". How were the clades defined? The authors state “there are several clades” – how did they define these clades? There do not seem to be any “clades” in Figure 5 and I wonder why/how one could describe genetic variability (or define clades) with a 203 bp segment. The best solution would be to remove the mention of “clades”. There are specific methods for defining genetic clusters (not necessarily high bootstrap support), and those methods were not used in the manuscript. This is a minor point, and it is only a suggestion for improvement, as many people write “clades” or “clusters” without such formal analysis. I was confused about their responses about this, so I thought I would mention it again.

**Response: We agree with this comment and removed “clades” as the reviewer’s suggestion. Please see lines 315-316.**

Reviewer #2:

Figure 6 – Please state what the box of dash line indicates.

**Response: We have provided information as the reviewer’s suggestion. Please see lines 1042-1043.**

Line 370 – Please add citation for statement about difference in sensitivity by nested-PCR and qPCR.

**Response: We have provided reference as the reviewer’s suggestion. Please see line 371.**

Line 373 – Please elaborate what is meant by “sequences were poor.”

**Response: When we request our sequences to company, they read two sides: forward and reverse and then send us both results. In some samples, forward was OK but reverse sequence has not read properly and some are opposite. ‘ Sequences were poor’ means that both sides were not properly read. Please see lines 371-372.**


Reviewer #1: The authors have not addressed the issue of PCR-positive/negative. They have included information about how many sequences were “selected” for analysis (209-210), but that is clearly not true, nor is it helpful resolving the issue raised previously. For example, clearly all 15 “successfully” sequenced A. phagocytophilum were included in Figure 2, and not “<5” as stated in the methods. Regardless, the issue is whether “unsuccessful” sequencing of a PCR product means that the sample is negative. The authors attempted to address these limitations in the discussion (paragraph beginning L369), but they have not addressed the issue of false-positivity.

I disagree with their statement that nested PCR is more sensitive and specific than qPCR (L369), and I would counter that nested PCR is more prone to false-positive results, and therefore less specific. The classical way to confirm conventional PCR (positivity and specificity) is to sequence the resulting amplicons. Otherwise, the samples should be considered putatively/potentially positive. The authors clearly state that 15 of 21 Anaplasma-positive (L269), 15 of 78 Bartonella-positives (L284), 2 of 5 Borellia-positives, etc were *successfully* sequenced. That is a significant proportion of PCR-positives. They mentioned that sequencing was not successful in the discussion (L431, L483). That is a huge "red flag" that those were not true positives.

The explanations the authors provide in the discussion (a “low density infection” or the fact that the primers do not match some Leptospira serovars) are unclear and create some additional problems.

The way to resolve this, as I suggested last time as well, is for the authors to state in the discussion that they considered PCR-positives to be truly positive, regardless of whether the amplicon could be sequenced, and note that this may overestimate the number of true positives, as some amplicons could not be sequenced. I suggest that they do not speculate that it is due to a “low density infection” (L431), and they do not imply that their PCR was inefficient (L483).

**Response: We agree with this comment and removed some parts that the reviewer mentioned. We have modified as the reviewer’s suggestion. Please see lines 371-376.**

Reviewer #1: L136 It is worth revising this more precisely. The virus is still called “severe fever with thrombocytopenia syndrome virus” and should still be called that. However, the official scientific name is Bandavirus dabieense. Just like the “striped field mouse” is Apodemus agrarius.

**Response: We have revised as the reviewer’s suggestion. Please see line 135.**

L255 correct the spelling of SFTSV

**Response: We have corrected it. Please see line 255.**

Reviewer #1: The authors have made many improvements to the manuscript. I think there are still a few minor points that should be corrected.

There are also some changes in the revision that I disagree with. I do not agree with the complete removal of information regarding unidentified rodents. It makes sense to concentrate the study on one species for the manuscript. However, I would expect that one would collect a variety of species using their stated trapping method. It now seems as though the authors only captured two species. This is suspicious and unlikely.

I do not agree with completely removing the pathogen-detection results from R norvegicus. My previous comments were intended to help clarify the data presentation and analysis, I was not suggesting the authors censor their data. Now it seems strange that the manuscript refers to pathogens in “rodents”, when, actually, the authors only present data from A agrarius. It is also strange that they state only 156 rodents were captured (which is untrue), list two species that they could identify, and then remove R norvegicus from the analysis.

There are two alternatives, as I see it: they state how many rodents were captured in total (175), and then state that this study focused on identifying human pathogens in the 151 A. agrarius (86%). Don't mention any other species at all.

Alternatively, they should return the results to how it was written originally: "A total of 175 rodents were captured and morphologically classified as follows: Apodemus agrarius (striped field mouse) (n = 151), Rattus norvegicus (Norway rat) (n = 5), and unknown (n = 19)." This was completely fine. My original comment was that including all rodents in the table was confusing. As there were only 5 brown rats, they could have simply stated these results in the respective sections (e.g., "Bartonella was detected in 3 brown rats").

**Response: We have modified as the reviewer’s suggestion. Please see lines 238-240.**

Reviewer #2: Please find below minor points and suggestions that may help.
Line 42 – Is the author’s suggestion for the ROK or worldwide as it known that rodents harbor various pathogens?

**Response: At this point, this indicates for the ROK. Please see lines 42-43.**

Line 148-149 – Sentence construction and word choice

**Response: We have modified as the reviewer’s suggestion. Please see line 149.**
Line 269 – “indicating that this pathogen was spread throughout the ROK.”

**Response: We have modified as the reviewer’s suggestion. Please see line 269.**

Line 345 – Suggest removing the word potentially as the pathogens characterized in the manuscript are zoonotic pathogens.

**Response: We have removed as the reviewer’s suggestion. Please see line 344.**

Line 407-410 – Please clarify sentence and the point the author is conveying.

**Response: We agree with this comment and have modified as the reviewer’s suggestion. Please see lines 402-403.**

Line 1020 – Sentence construction and suggest the use of the word symbol instead of markings.

**Response: We have modified as the reviewer’s suggestion. Please see line 1021 and new figure 1.**
