# Supplementary material for: Molecular surveillance of zoonotic pathogens from wild rodents in the Republic of Korea
Source: PLoS Negl Trop Dis. 2024 Jul 8;18(7):e0012306. doi: 10.1371/journal.pntd.0012306 (PMC11257403; doi:10.1371/journal.pntd.0012306)
Supplement: Attachment — Submitted filename: Reviewer Comments.docx [file pntd.0012306.s003.docx]

**Reviewer Comments**

**Minor**

Line 48 – The statement that rodents live anywhere in the world is incorrect.

Line 65 – Add other transmission routes through which indirect transmission of zoonotic diseases from rodents occur.

Line 127 – Climate change is not a qualifier for heavy rainfall and flooding to occur.

Line 166 – It would be informative to know how long the capture duration was a particular site.

Line 169 – How were captured rodents identified and were identification of rodents confirmed through molecular identification methods?

Line 172 – Write out abbreviation of SST.

Line 203 – State reference sequence accession numbers used to determine similarity.

Line 203 - State which tool was used to determine sequence similarity.

Line 217 – It would be informative to know if information about the rodents such as age and sex was collected during capturing of rodents.

Line 239 – Sentence not appropriate in Results should be moved to Discussion section.

Table 3 – Missing description of the hyphen.

**Major**

I suggest the manuscript undergoes major revisions to improve the writing to address sentence construction and grammar, below is a limited list of examples of concern.
Line 49 – Sentence construction and word choice.
Line 220 – Sentence construction and word choice.
Line 228 – Sentence not needed.
Line 231 - Sentence construction and word choice.
Line 238 – SFTSV wasn’t co-infected with *Bartonella* spp. rather the rodent was co-infected with SFTSV and *Bartonella* spp.

Line 204 – How were sequences of different length treated and used for sequence alignment and phylogenetic analysis in relationship to study obtained and reference sequences?

Figures 2, 3, 4, 5, 6, and 7 – The rodent species and the province of where the sample was collected needs to be added to the accession number.

Figure 2, 3, 6 – Bootstrap values are missing from smaller branches.

Figure 7 – Sample names aren’t uniform to what was used in other figures containing phylogenetic trees.

Table 2 - Since no analysis were performed on unknown samples and they aren’t discussed in manuscript it be removed from the table.
